# A transposon-based genetic marker for conspecific identity within the *Bactrocera dorsalis* species complex

**DOI:** 10.1038/s41598-023-51068-2

**Published:** 2024-01-22

**Authors:** Grazyna J. Zimowska, Nirmala Xavier, Masroor Qadri, Alfred M. Handler

**Affiliations:** 1 U.S. Department of Agriculture, Center for Medical, Agricultural, and Veterinary Entomology, Agricultural Research Service, 1700 SW 23rd Drive, Gainesville, FL 32608 USA; 2 Entomology and Nematology Department, University of Florida, Gainesville, FL 32611 USA

**Keywords:** Evolutionary genetics, Molecular evolution, Phylogenetics, Speciation, Taxonomy, Evolution, Genetics, Molecular biology

## Abstract

Here we describe a molecular approach to assess conspecific identity that relies on the comparison of an evolved mutated transposable element sequence and its genomic insertion site in individuals from closely related species. This was explored with the IFP2 *piggyBac* transposon, originally discovered in *Trichoplusia ni* as a 2472 bp functional element, that was subsequently found as mutated elements in seven species within the *Bactrocera dorsalis* species complex. In a *B. dorsalis* [Hendel] strain collected in Kahuku, Hawaii, a degenerate 2420 bp *piggyBac* sequence (*pBac*^*Bd*-Kah^) having ~ 94.5% sequence identity to IFP2 was isolated, and it was reasoned that common species, or strains within species, should share the same evolved element and its precise genomic insertion site. To test this assumption, PCR using primers to *pBac*^*Bd*-Kah^ and adjacent genomic sequences was used to isolate and compare homologous sequences in strains of four sibling species within the complex. Three of these taxa, *B. papayae*, *B. philippinensis*, and *B. invadens*, were previously synonymized with *B. dorsalis*, and found to share nearly identical *pBac*^*Bd*-Kah^ homologous elements (> 99% nucleotide identity) within the identical insertion site consistent with conspecific species. The fourth species tested, *B. carambolae*, considered to be a closely related yet independent species sympatric with *B. dorsalis*, also shared the *pBac*^*Bd*-Kah^ sequence and insertion site in one strain from Suriname, while another divergent *pBac*^*Bd-*Kah^ derivative, closer in identity to IFP2, was found in individuals from French Guiana, Bangladesh and Malaysia. This data, along with the absence of *pBac*^*Bd*-Kah^ in distantly related *Bactrocera*, indicates that mutated descendants of *piggyBac*, as well as other invasive mobile elements, could be reliable genomic markers for common species identity.

## Introduction

Molecular genetic markers based upon DNA sequence variation represent one of the more powerful tools to assess the evolutionary relationship between related insect species, and is essential for distinguishing closely related species that cannot be unambiguously defined by morphological, chemical or behavioral criteria^1^. However, DNA-based distinctions also have limitations based upon sequence length, nuclear or mitochondrial origin, inherent variability, importance to organismal viability, and for closely related species not all genetic data may be in agreement for defining heterospecific species. This is exemplified for tephritid fruit flies within the *Bactrocera dorsalis* species complex that encompasses 100 or more geographically dispersed species^2–4^. Among these, the source species for the complex, the oriental fruit fly *Bactrocera dorsalis s.s.* (Hendel), and the closely related sibling species, *B. papayae* (Drew and Hancock) and *B. philippinensis* (Drew and Hancock) were originally defined as independent species based upon morphometric analysis^5^, that subsequently included the more recently discovered African species, *B. invadens*^6^. However, the abundance of genetic and chemoecological evidence for synonymization of the four species as *B. dorsalis s.s.* is now generally accepted, but does not unambiguously include their relationship to another close relative, the carambola fruit fly, *B. carambolae* (Drew and Hancock)^7–10^.

Distinctions between *B. dorsalis* and *B. carambolae* have been based on several criteria including morphological differences in wing shape^11^, aedeagus length and abdominal color patterns^12^; differences in cuticle and pheromone chemistry profiles^13^; and mating compatibility based on assortative mating under semi-natural conditions^14^. Genetic analyses of this relationship, in addition to the synonymized taxa, have centered on sequence comparisons of several genetic loci, microsatellite regions, and their mitochondrial genomes. The most extensive study comparing independent genetic loci compared sequences of six loci, including *cox1*, *nad4-3′, CAD, period*, ITS1, and ITS2, from approximately 20 individuals collected from 16 sample sites^7^. This study, at a minimum, failed to resolve a distinction between the subsequently synonymized *B. dorsalis* taxa, but did conclude that *B. carambolae* was a distinct monophyletic clade. Further support for independent taxa was also provided by the whole mitochondrial genome sequence comparisons between nine strains identified as *B. carambolae* and five strains identified as *B. dorsalis* based upon specific polymorphisms in the respective taxa^15^. However, this relationship was less well defined in an extensive study using eight microsatellite DNA markers to define the relationships among seven geographically separated *B. carambolae* strains and three *B. dorsalis* strains in Southeast Asia^16^. Here genetic diversity was observed among the *B. carambolae* strains, and between *B. dorsalis*, but enough overlap was observed in a subset of populations to indicate the presence of hybrid groups suggesting incomplete reproductive isolation between the taxa. Yet another study found no genetic structure in haplotype diversity between the two species after sequencing 765 bp of the mitochondrial COI gene in 1,601 flies from 19 geographically diverse populations^17^. This observation more clearly indicated consistent genetic exchange occurring between the two taxa, likely the result of incomplete reproductive isolation. In an earlier comparison of mitochondrial ribosomal DNA in 19 *Bactrocera* species, and tRNALeu and flanking COI /COII regions in 27 species, *B. carambolae* was positioned closer to *B. dorsalis* than *B. philippinensis* in common clades^18,19^, which is more consistent with *B. carambolae* sharing species identity with both. In addition, a cytogenetic analysis of polytene and mitotic chromosomes from synonymized *B. dorsalis* complex species and *B. carambolae* failed to find any apparent distinction between any of the taxa^20^.

While defining the relationship of these closely related *Bactrocera* species is important to expanding our knowledge of the evolutionary relationships within the *B. dorsalis* species complex, these taxa are also among the most agriculturally and economically important invasive pest species in the world. Thus, a determination of whether they are conspecific or independent has significant relevance to population control strategies, and the implementation of trade barriers^9,21^. These considerations add impetus to the need to resolve their actual relationship more precisely that has yet to be achieved conclusively for *B. dorsalis* and *B. carambolae*, for which the use of a unique genetic marker described here may improve upon.

Our initial interest in resolving the identification of these species within the *B. dorsalis* complex arose after our initial discovery of *piggyBac*-like transposable elements in *B. dorsalis*, that were nearly identical to the original IFP2 *piggyBac* transposon discovered in a cabbage looper moth, *Trichoplusia ni*, cell line^22^. We used this element to create a vector for germ-line transformation of *B. dorsalis*, and genomic Southern blot analysis of transformant lines revealed non-vector *piggyBac*-like sequences^23^. Subsequent PCR studies identified high identity *piggyBac* elements in this and several other *B. dorsalis* strains, nine additional species within the *B. dorsalis* species complex, and in several members of the *B. zonata* and *B. tryoni* complexes^24,25^. While all of these internal sequences shared high levels of nucleotide and amino acid identity, most of the consensus reading frames included frameshift mutations, and while two non-identical elements had complete open reading frames, from *B. minuta* and *B. zonata*, neither were proven to encode functional transposase.

Nevertheless, it was realized that the nuclear genomic profile of mutated descendants of an invasive transposon family, or families, could provide molecular markers for species-specificity, though much of this analysis had focused on transposon display of retrotransposons in plant species^26,27^, as well as analysis of unique miniature inverted repeat transposable elements (MITES)^28^. We postulated, however, that common species identity could also be inferred by nearly identical, albeit significantly mutated, transposon sequences within a common genomic insertion site in different individuals or strains. It was reasoned that while it was possible for an identical functional transposon, having an insertion site specificity, to be discovered in a common genomic site in different reproductively isolated species, it would be highly unlikely for the same element to follow nearly identical paths of mutation accumulation in these species as a neutral element after initial inactivation of autonomous mobility. In addition, the identity of common proximal genomic sequences was expected to provide added support for relatedness, and the level of transposon sequence degeneracy relative to the functional element could provide a time estimation for the initial invasion of the ancestral transposon into the species, presumably by inter-species horizontal transfer^29,30^.

To test the possibility that a complete mutated *piggyBac* descendant transposon, and its proximal genomic insertion site sequences, could be a marker for common species identity, we isolated a complete, albeit mutated, *piggyBac*-like transposon from the wild type Kahuku Hawaiian strain of *B. dorsalis* (*pBac*^*Bd*-Kah^). To identify homologs of this element, whose insertion site and sequence identity could be an indicator of phylogenetic relatedness, PCR was performed with internal *piggyBac* primers and proximal genomic primers in other *Bactrocera* strains and species. The initial preliminary tests were on Tanzanian flies morphologically similar to *B. dorsalis*^31,32^, but later defined as an independent species, *B. invadens*^6^, which provided the first molecular evidence for them being a common species^33,34^. Further validation for this approach came from repetition of this analysis and expansion to include additional *B. dorsalis* strains, strains of all the synonymized species, and more distantly related species considered to be monophyletic, but known to harbor *piggyBac*-like elements including four strains of *B. carambolae*^24^. Notably, *B. carambolae* is the most closely related species to *B. dorsalis* considered to be monophyletic, but whose actual relationship has not been definitively determined for all geographically distinct strains^16,17^.

## Methods

### Insects

The *Bactrocera* species analyzed for this study were all verified by taxonomists and are listed in Table 1 with their country of origin, and region/locale collected if known. References to source material, collections or reports of previous phylogenetic analysis are listed, and for those strains in which the *pBac*^*Bd*-Kah^ homolog element has been isolated the GenBank accession number has been provided.

**Table 1:** *Bactrocera* species screened for homologs of the *B. dorsalis*-Kahuku *piggyBac* element.

Host species	Country/state	Locale/region/lab (strain)	GenBank accession #	References^1^
*pBac*^*Bd*-Kah^ homolog identified				
*B. carambolae*	Suriname	Paramaribo	OQ718444	a, b, c
	French Guiana	wild (unknown)	OQ718445	d
	Malaysia	Sabah	OQ718446	d
	Bangladesh	Chittagong	OQ718447	d
*B. dorsalis*	Hawaii-Oahu	Kahuku (Kah)	OQ718442	e
	California	Genetic sexing strain (GSS)	OQ718443	a
*B. invadens*	Kenya	Lab strain	OQ718448	a, c
	Tanzania	Dar Es Salaam (DES2)	OQ718449	f
	Tanzania	Kilamanjaro (KA)	OQ718450	f
*B. papayae*	Australia	Wild (unknown)	OQ718451	a, b, c
	Malaysia	Serdang	OQ718452	a, c
*B. philippinensis*	Philippines	wild1 (unknown)	OQ718453	a, c
	Philippines	Guimaras Island	OQ718454	a, c
*pBac*^*Bd*-Kah^ homolog not identified				
*B. oleae*	Greece	Lab strain		a
*B. tryoni*	Australia	Narara		a
*B. zonata*	Mauritius	Lab strain		a
*Zeugodacus cucurbitae*	Hawaii	Genetic sexing strain		a

^1^References for initial collections, or collection that provided insect samples, and previous phylogenetic analysis of the respective strains: (a) IAEA Insect Pest Control Laboratory Tephritid species collection maintained since 2011; (b) Handler et al. (2008); (c) Schutze et al. (2015); (d) San Jose et al. (2018); (e) University of Hawaii (S.D. McCombs); (f) USDA-APHIS-PPQ (T. Holler).

### Isolation of full-length *piggyBac* elements from the *B. dorsalis* Kahuku wild type strain

**Figure 1:**
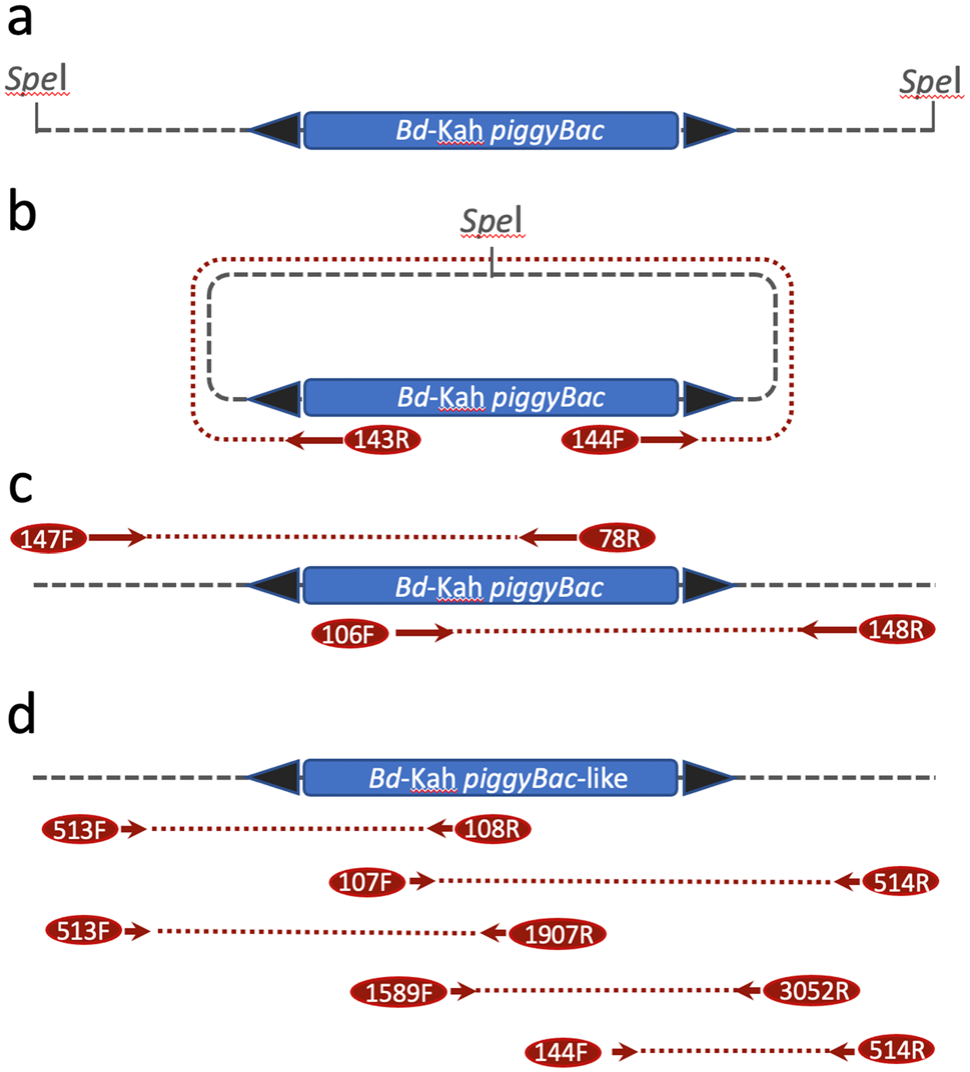
Illustration of the steps for isolating full-length *piggyBac* elements in the *B. dorsalis* Kahuku strain by inverse PCR and the use of adjacent genomic sequences for PCR isolation and sequence analysis of homologous elements in closely related *Bactrocera* species. (**a**) The *Spe*I restriction enzyme, that does not have a restriction site within IFP2 *piggyBac*, is used to digest whole genomic DNA; (**b**) circularization of *Spe*I digested DNA by self-ligation and use of *piggyBac* terminal sequence primers in opposite orientation to sequence adjacent 5′ and 3′ genomic insertion site DNA; (**c**) genomic and internal *piggyBac* primer pairs used to generate and sequence overlapping amplicons within the *pBac*^*Bd*-Kah^ element extending out to the 5′ and 3′ genomic DNA; and d) additional primer pairs used to generate overlapping sequences in homologous *pBac*^*Bd*-Kah^-like elements in *B. dorsalis* complex species for sequence isolation and verification. Blue dashed lines represent genomic DNA (not to scale); arrows represent forward (F; >) and reverse (R; <) primers sites; and maroon dotted lines represent PCR amplicons (not to scale; see Table S1 for primer sequences).

To isolate full-length, though potentially non-functional, *piggyBac* elements from the *B. dorsalis* Kahuku wild type strain, inverse PCR was performed by first digesting ~ 1 µg genomic DNA, isolated with DNAzol (Molecular Research Center), with the *Spe* I restriction endonuclease, which does not cut within the functional IFP2 sequence, for 4 h with subsequent ethanol precipitation (Fig. 1). The digested fragments were resuspended in dilute conditions (60 µl) and circularized by overnight ligation at 12 °C. The Expand High Fidelity PCR System (Roche) was then used for outward long-template PCR from the *piggyBac* 5′ and 3′ arms using reverse (AH143R) and forward (AH144F) primers (Table S1), respectively, to amplify terminal sequences and the adjacent genomic insertion sites sequences using the cycling conditions: initial denaturation for 2 min at 95 °C; 40 cycles denaturation for 30 s at 95 °C, annealing for 30 s at 55 °C, extension for 2 min at 68 °C; and a final extension for 7 min at 68 °C. PCR products, separated by 1% agarose gel electrophoresis, greater than 300 bp in length (the expected length of amplified *piggyBac* sequences) were isolated by gel elution and ligated into the TOPO TA cloning vector (Invitrogen) and sequenced from both ends using M13 forward and reverse primers (Macrogen). DNA sequence analysis was performed with Lasergene (DNASTAR) and Geneious (Biomatters) software. To isolate internal sequences for the individual full-length specific *piggyBac* element identified (since multiple highly similar elements exist in the genome;^24^) direct PCR was initially performed using anchor primer sites in the proximal genomic DNA with internal primer sites based on the IFP2 sequence, that included primer pairs for overlapping amplicons generated by AH147F/AH78R for the 5′ sequences and AH106F/AH148R for the 3′ sequences. Cycling conditions consisted of initial denaturation for 2 min at 95 °C; 35 cycles of denaturation for 30 s at 95 °C; annealing for 30 s at 58 °C; extension for 2 min at 72 °C; and a final extension for 7 min at 72 °C. De novo assembly of overlapping amplicons then provided a complete consensus sequence that allowed additional primer design for sequence verification of the Kahuku element (*pBac*^*Bd*-Kah^) and *Bactrocera* homologous elements using several internal *piggyBac* sites with the genomic primers.

### Isolation of *pBac*^*Bd*-Kah^ homologs from *Bactrocera* strains

For isolation of additional elements homologous to *pBac*^*Bd*-Kah^ the AH513F forward (427 bp 5′ upstream) and AH514R reverse (371 bp 3′ downstream) genomic primers were used with internal primers to isolate *pBac*^*Bd*-Kah^ homologs in other *Bactrocera* species and strains. The initial strategy for most strains was to use the AH513F/AH108R and AH107F/AH514R primer pairs for overlapping 1096 bp 5′ and 2192 bp 3′ amplicons, respectively, generating 3.2 kb concatenated sequences in additional strains of *B. dorsalis*, *B. invadens*, *B. carambolae*, *B. papaya*e and *B. philippinensis*. Additional primer pairs were used for sequence verification and for instances where the initial PCR reactions did not amplify adequately or accurately, possibly due to nucleotide substitutions or degraded DNA in preserved specimens (Fig. 1; see Table S1 for primer sequences and binding sites).

PCR amplifications for these strains were performed on genomic DNA samples prepared with either DNAzol (Molecular Research Center), the DNeasy Blood and Tissue Kit (Qiagen) or the MasterPure DNA Purification Kit (Lucigen) using the Q5 or Phusion High Fidelity PCR Systems (New England Biolabs) under cycling conditions of: 98 °C for 2 min initial denaturation, and 35 cycles of 98 °C denaturation for 30 s, 58 to 68 °C annealing for 30 s, 30 s to 2 min extension at 72 °C, with a final 72 °C extension for 5 min. Amplified DNA was visualized on 0.8 to 1% agarose gels, isolated by gel elution and either sequenced directly using PCR primers or subcloned into the TOPO TA cloning vector and sequenced using M13 forward and reverse primers, with most PCR products sequenced on both strands. Forward (F) and reverse (R) primers used for amplification and sequencing of *piggyBac* and genomic insertion site sequencing are provided in Table S1.

### Sequence analysis and phylogenetic comparisons

DNA sequence analysis, alignments, and de novo assembly of the *pBac*^*Bd*-Kah^ homologous sequences from other *B. dorsalis* strains and *Bactrocera* species, and their genomic insertion sites, were performed using ClustalX^35^ and Clustal Omega^36^ within Geneious Prime software (Biomatters) and phylogenetic analysis was performed on MEGA_X^37,38^. Sequence pair distances were determined from the Clustal Omega multiple sequence alignments. Maximum Likelihood (ML) phylogenetic analysis using the General Time Reversible model^37,39^ with 1000 bootstrap replicates for branch support^40^ was performed on concatenated *pBac*^*Bd*-Kah^ and *pBac*^*Bd*-Kah^ homolog sequences, with and without adjacent genomic insertion site sequences.

The Tamura-Nei^41^ evolutionary model was used to calculate the genetic distance divergence time (*t*) between the ancestral *piggyBac* IFP2 functional element and the descendant *pBac*^*Bd*-Kah^ homologous sequences using the formula *t* = *K/v*, where* K* is the genetic distance and *v* is the neutral substitution rate. The single-nucleotide mutation rate for *Drosophila melanogaster* was used, calculated as 0.0058 mutations per site per million generations by Haag-Liautard et al. ^42^, and an estimated generation time of 30 days (parental egg/embryo to progeny egg/embryo stage) was used to convert the number of generations to years.

## Results

### Isolation of the *piggyBac*^*Bd*-Kah^ element

**Figure 2:**
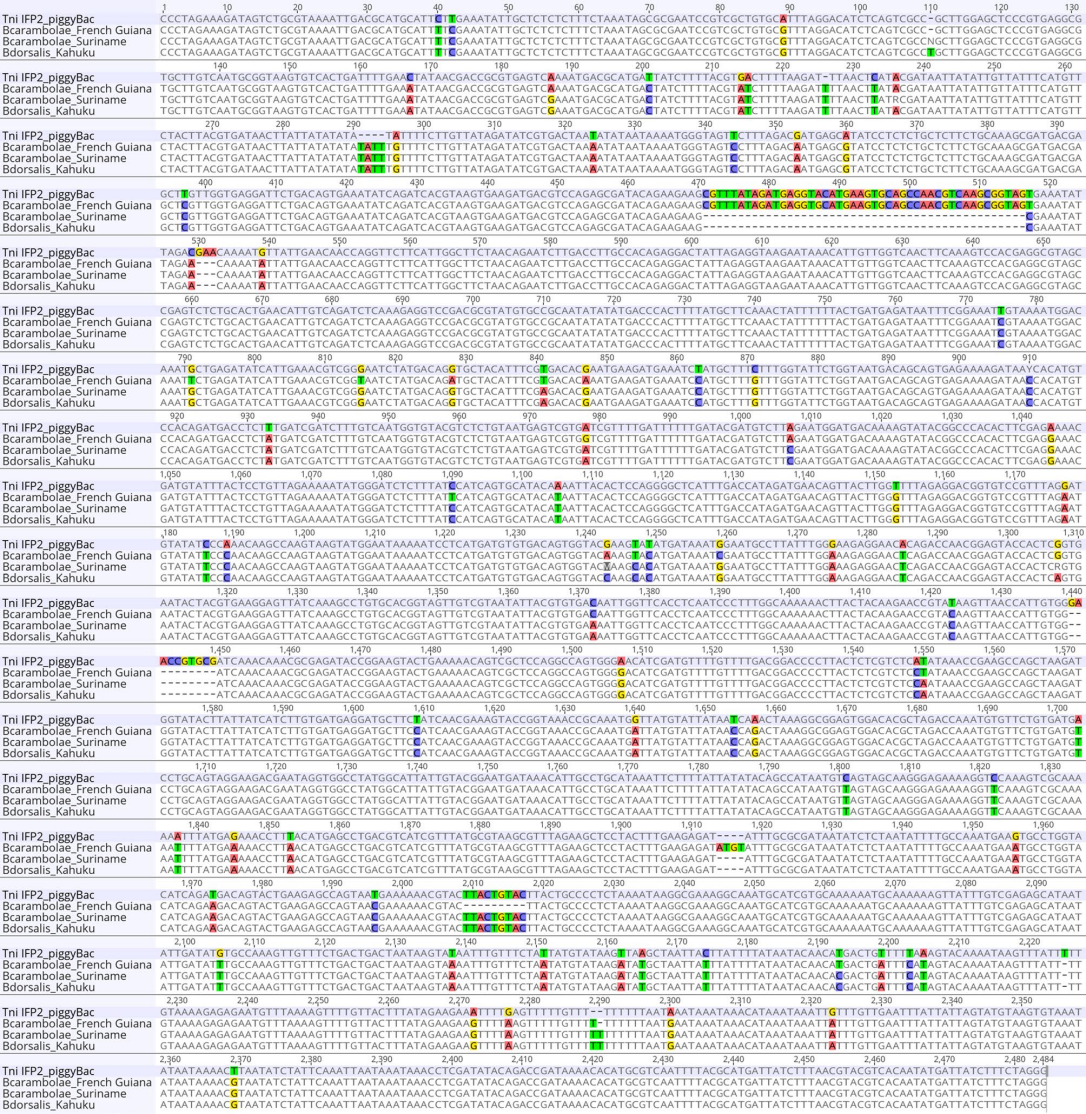
Clustal Omega nucleotide sequence alignment of the full-length *T. ni* IFP2 *piggyBac* transposon sequence (2472 bp), the *B. dorsalis pBac*^*Bd*-Kah^ element (2420 bp), and *pBac*^*Bd*-Kah^ homologs isolated from two *B. carambolae* strains. The *B. carambolae*-Suriname element is nearly identical to the *pBac*^*Bd*-Kah^ element and homologs isolated from synonymized taxa, while the *B. carambolae*-French Guiana element, having high identity to the *B. carambolae*-Malaysia and Bangladesh sequences (see Fig. 3 and Fig. S1), is significantly diverged.

To isolate complete *piggyBac* elements from *B. dorsalis*, an inverse PCR approach was taken to amplify the terminal sequences of both arms of full-length elements and adjacent insertion site genomic DNA. This was performed in a wild type strain collected in the Kahuku region of Oahu, Hawaii (by S.D. McCombs, University of Hawaii) in which full-length *piggyBac* elements and partial sequences were inferred by DNA hybridization and PCR sequence analysis^23,24^ (Fig. 1). From several PCR products sequenced, only one included the expected 5′ and 3′ inverted terminal repeat (ITR) *piggyBac* sequences that was inserted into approximately 1300 bp of circularized genomic DNA. Internal sequences for the specific *piggyBac* element was performed by direct PCR using primer sites in the proximal genomic DNA with internal primer sites resulting in overlapping amplicons. The concatenated sequences generated a 3585 bp sequence that included a 2420 bp *B. dorsalis* Kahuku *piggyBac* sequence, *pBac*^*Bd*-Kah^, and 1165 bp of adjacent genomic insertion site DNA (608 bp 5′ and 557 bp 3′genomic DNA), derived from alignment to the 2472 bp IFP2 element (Fig. 2). Subsequent PCR was performed on *pBac*^*Bd*-Kah^ and *Bactrocera* homologous elements using several internal *piggyBac* sites with the genomic primer sites encompassing a total length of 3,218 bp.

**Figure 3:**
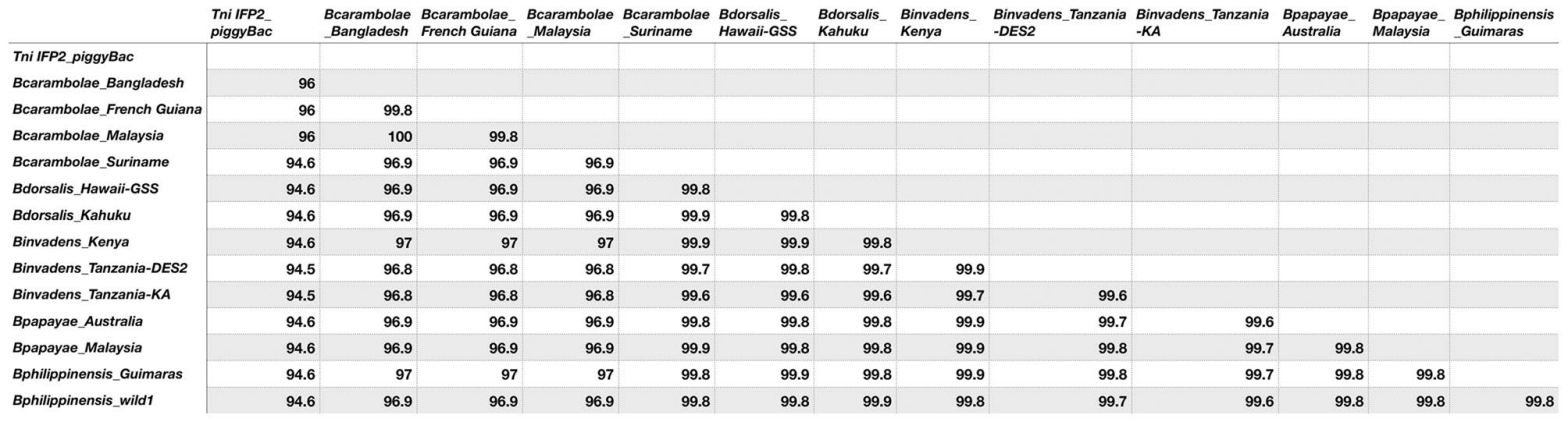
Percent identity matrix between the full-length 2472 bp *T. ni* IFP2 *piggyBac* sequence and 2419 bp to 2420 bp *pBac*^*Bd*-Kah^ homolog nucleotide sequences isolated from indicated strains from *B. dorsalis* complex species. The distance matrix was generated by a Clustal Omega multiple sequence alignment (see Fig. S1).

Clustal Omega alignment^36^ of the *pBac*^*Bd*-Kah^ 2420 bp element and the 2472 bp IFP2 element (Fig. 2) revealed 63 nt deleted nucleotides, 8 nt insertions and 66 nt substitutions in *pBac*^*Bd*-Kah^ relative to IFP, resulting in a nucleotide identity of 94.6% (Fig. 3). Since several indels within the consensus transposase reading frame from nts 329–2113 in IFP2 are likely to result in a truncated protein, it is presumed that the *pBac*^*Bd*-Kah^ element and its homologs are non-functional in terms of autonomous self-mobilization. However, *pBac*^*Bd*-Kah^ mobility facilitated *in trans* by functional transposase remains a possibility since the critical 5′ and 3′ terminal and sub-terminal inverted repeat sequences are intact. But genomic transposition remains uncertain since additional terminal sequence within ~ 100 bp of each terminus may be required^43^, and several nucleotide substitutions exist within the 5′ terminus of *pBac*^*Bd*-Kah^. This element, and all *Bactrocera* homologs described below, are bounded by an intact duplication of the canonical TTAA genomic insertion site for *piggyBac* elements consistent with it being a descendant of an autonomous functional element.

### *piggyBac*^*Bd-*Kah^ homologs in species closely related to *B. dorsalis* Kahuku

The complete sequence of *pBac*^*Bd*-Kah^ and its proximal genomic DNA allowed us to determine whether the same element existed in closely related species, some accepted to be conspecific with *B. dorsalis s.s.* This was addressed by isolating homologous elements in 8 other sibling species using proximal genomic and internal *piggyBac* primers (Fig. 1, Table S1). In addition to *B. dorsalis*, at least two of the more distantly related species surveyed, *B. zonata* and *B. tryoni*, are known to have multiple full-length and/or partial *piggyBac* sequences^24,25^. Therefore specific isolation of *pBac*^*Bd*-Kah^ homologs required genomic primer anchors that could be paired with overlapping internal primers that included primer pairs 513F/108R, 513F/1907R, 107F/514R, 1589F/3052R, and 144F/514R (Fig. 1, Table S1).

PCR amplification of homologous *pBac*^*Bd*-Kah^ sequences with these primer pairs were aligned to create full-length consensus sequences in at least two different strains of the synonymized species *B. dorsalis*, *B. papayae*, *B. philippinensis*, and *B. invadens*, and the non-synonymized species, *B. carambolae*. Identifiable PCR products were not amplified from any of the primer pairs in the more distantly related species *B. zonata*, *B. tryoni*, *B. oleae*, and *Zeugodacus curcurbitae*, although internal *piggyBac* sequences with high identity were previously isolated in *B. zonata* and *B. tryoni*^24^.

### Nucleotide sequence comparisons of *piggyBac*^*Bd*-Kah^ homologs

Consistent with *pBac*^*Bd*-Kah^, the sequence length of homologs of all strains from the four synonymized *B*. *dorsalis* species was identical at 2420 bp, except for a 2419 bp element in *B. invadens*_Kenya. The percent identities for the *piggyBac* element among these strains from a Clustal Omega alignment ranged from 99.6 to 99.9%, while identity to the IFP2 element ranged from 94.5 to 94.6%, sharing nearly all the same major signature differences including 3-bp, 4-bp, 10-bp and 46-bp deletions and a 1-bp and 4-bp insertions, with a majority consensus for 66 nucleotide substitutions (Figs. 2, 3 and S1). Notably, the *B. carambolae*_Suriname 2420 bp element shared similar identities with the *B. dorsalis* synonymized species elements, while the *B. carambolae* 2459 bp elements from Bangladesh, French Guiana and Malaysia, sharing greater than 99.8% identity to each other, were more diverged, ranging in identity to the other strains from 96.8 to 97% (Fig. 3). Indeed, the three strains were more closely related to IFP2, having the same identities of 96%, primarily due to the absence of a consensus 46-bp deletion found in the other strains, but they shared the 3-bp, 4-bp and 10-bp deletions, the 1-bp and 4-bp insertions and 59 of the nucleotide substitutions, in addition to having a unique 4-bp insertion, 9-bp deletion and 7 substitutions. Since the 46-bp deletion is not present in IFP2, it may have occurred more recently than the majority of other common sequence variations, or occurred independently in a reproductively isolated sub-population. A continued survey of *pBac*^*Bd*-Kah^ sequences will be required to assess the frequency and relevance of the Bangladesh, French Guiana and Malaysia variants (and possibly others) from two geographically distinct regions, but given their occurrence in three of four strains tested, this may reflect the broad genomic diversity found in previous studies of *B.carambolae* natural populations^16,17^.

### Phylogenetic analysis of *piggyBac*^*Bd*-Kah^ homologous sequences

**Figure 4:**
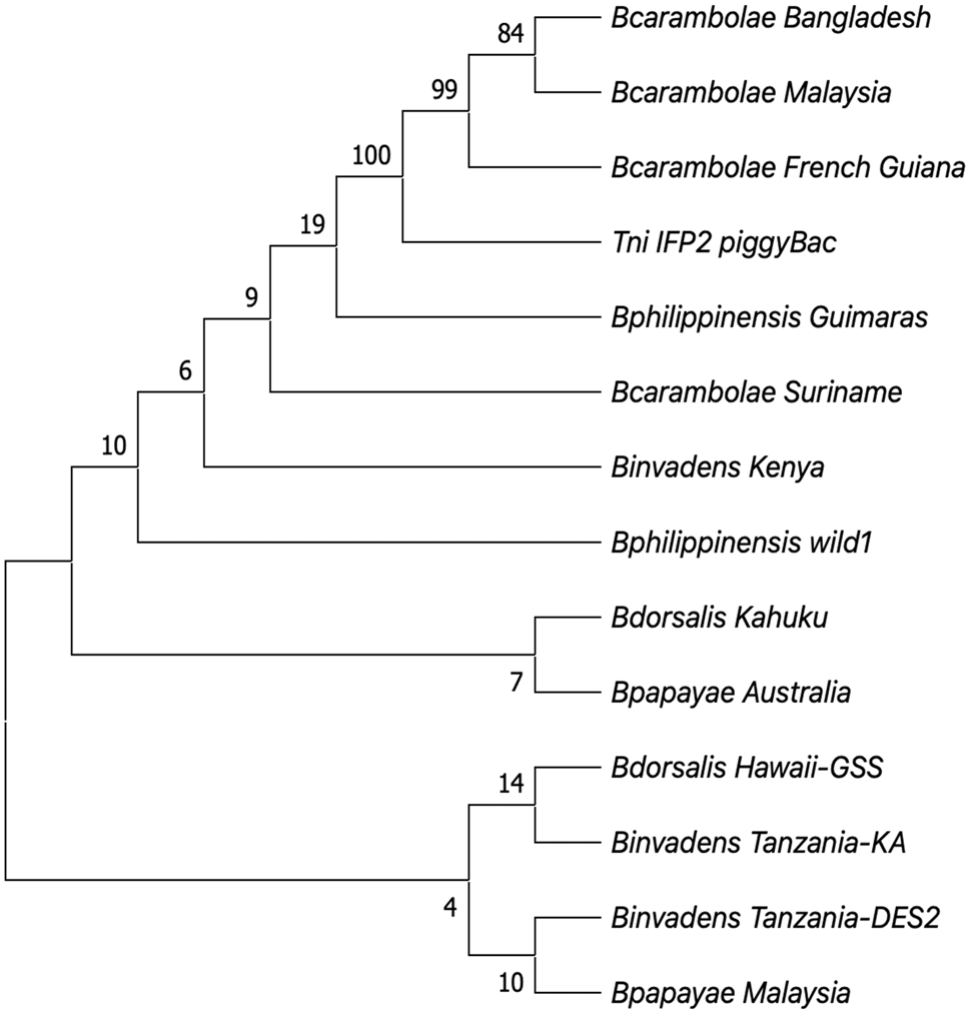
Use of the Maximum Likelihood method and General Time Reversible model for the phylogenetic analysis of nucleotide sequences aligned by Clustal Omega (see Fig. S1) including the full-length *T. ni* IFP2 *piggyBac* transposon (2472 bp) and descendant *pBac*^Bd-Kah^ homologs (2419–2459 bp) isolated from 13 strains from five *Bactrocera dorsalis* complex species. The bootstrap consensus tree is inferred from 1000 replicates where the percentage of replicate trees in which the associated taxa clustered are shown above the branches. Initial trees for the heuristic search were obtained automatically by applying Neighbor-Join and BioNJ algorithms to a matrix of pairwise distances estimated using the Maximum Composite Likelihood (MCL) approach. A discrete Gamma distribution was used to model evolutionary rate differences among sites (5 categories (+ *G*, parameter = 3.4437)). This analysis involved 14 nucleotide sequences. There were a total of 2487 positions in the final dataset. Evolutionary analyses were conducted in MEGA_X.

MEGA_X was used to perform maximum-likelihood phylogenetic analysis using the General Time Reversible model of the evolutionarily neutral *pBac*^*Bd*-Kah^ homologous sequences^37^. The resultant tree after 1000 bootstrap replications was consistent with minimal levels of nucleotide divergence, supported by low bootstrap values, among the synonymized *B. dorsalis* taxa and the *B. carambolae*-Suriname strain (Fig. 4). Divergence was most strongly supported for the clade including the *B. carambolae*-Bangladesh, French Guiana and Malaysia strains, that clustered more significantly with IFP2 *piggyBac*.

 Using the *t* = *Kν* formula^41,42,44^ divergence times of approximately ~ 5 million generations, translated to ~ 0.4 Myr divergence based on 30 days/generation, were calculated for all the sequences relative to their functional ancestral element, expected to be homologous to *T. ni IFP2_piggyBac* (Table S2). However, the 95% confidence intervals range from ~ 2 M to ~ 14 M for generations reflect a relatively high uncertainty in *ν*.

### Comparison of genomic insertion site sequences of *piggyBac*^*Bd*-Kah^ homologs

Sequence comparisons were also made for the respective *pBac*^*Bd*-Kah^ homologous sequence insertion sites, that were expected to be highly similar based upon their successful use for PCR isolation. Using sequences from PCR with primer pairs 513F/108R and 513F/1907R for the 5’ genomic DNA and 107F/514R and 144F/514R for the 3’ genomic DNA, the *pBac*^*Bd*-Kah^ homolog DNA was deleted in addition to one of the duplicated TTAA tetranucleotide sequences (that are duplicated during the transposition process). The remaining 5’ and 3’ genomic sequences were concatenated resulting in 793 bp to 797 bp sequences that were aligned by Clustal Omega (Fig. S2A). Unlike the *pBac*^*Bd*-Kah^ homolog sequences, the insertion site sequence identities for the *B. dorsalis* synonymized species and the *B. carambolae* strains were generally greater than 99%, except for *B. carambolae*-Malaysia, that averaged ~ 1% less (Fig. S2B).

Blastn queries with the *pBac*^*Bd*-Kah^ insertion site to *Bactrocera* (taxid: 27456), Insecta (taxid: 50557) and nr databases all yielded similar high identity hits to tyrosine kinase, unspecified nucleases, and non-annotated sequences from several *Bactrocera* species. Conceptual translation of the sequences did not yield any meaningful reading frames.

## Discussion

In this study we report the identification of a mutated non-autonomous *piggyBac* transposable element, based upon an interrupted transposase open reading frame, in the genome of a wild type Hawaiian strain of *B. dorsalis*. Isolation of this element used an inverse PCR approach that identified the adjacent genomic insertion site DNA sequences that were subsequently used in direct PCR to isolate the complete homologous element in other strains and species in which it might exist. This analysis was performed in the sibling species now accepted to be synonymized with *B. dorsalis s.s.*, including *B. papayae, B philippinensis* and *B. invadens*^7,9,10^, in addition to testing more distantly related species within and beyond the *Bactrocera dorsalis* complex. Our results are consistent with the notion that a non-functional mutated transposon, having a unique sequence that evolved from the functional element, could be a reliable genetic marker for common species identity. Specifically, in the taxa now considered to be *B. dorsalis s.s*., the degenerate 2,420 bp homologous *piggyBac* elements that share the identical genomic insertion site have greater than 99.6% identity to one another, and no greater than 94.4% to 94.5% identity to the functional IFP2 element that, presumably, they descended from. Their common species identity is also reflected in the lack of phylogenetic nodal support for significant nucleotide divergence among the taxa, and by the 99.1% to 100% identity range for the 793 bp to 797 bp adjacent 5’ and 3’ insertion site genomic DNA. Neither the consensus degenerate *pBac*^*Bd*-Kah^ element nor the genomic insertion site DNA could be amplified using a series of primer pairs in species considered to be more distantly related to *B. dorsalis*, including *B. zonata* and *B. tryoni*, in which *piggyBac* sequences were previously discovered^24,25^, indicating the absence of *pBac*^*Bd*-Kah^ and divergence of its genomic insertion site. However, while the high level of identity and common insertion sites for *pBac*^*Bd*-Kah^ in the *B. carambolae*-Suriname strain and the *B. dorsalis* taxa is consistent with their common relationship, phylogenetic comparisons of *B. carambolae* to other *Bactrocera* suggest that another Suriname strain is distinct from *B. dorsalis*^45^.

It is also notable that divergence of the South American *B. carambolae*-Suriname strain from the Asian strains bears consistency with the studies of Boykin et al.^7^ that found within the resolved *B. carambolae* species, that all the Suriname samples uniquely formed a well-supported monophyletic sub-clade distinct from the southeast Asian samples. While it was suggested that this may have resulted from a genetic bottleneck related to its invasion into South America, the French Guiana strain in our study, which neighbors Suriname, remains closely associated with the Asian strains from Bangladesh and Malaysia indicating divergence within a common geographic region. However, the *pBac*^*Bd*-Kah^ homologous elements found in the Bangladesh, French Guiana and Malaysia strains of *B. carambolae* exhibited greater identity to IFP2 represented by distinct nucleotide substitutions and indels not present in the Suriname strain nor the other *B. dorsalis* taxa, including a common 46-bp deletion. Together, this data reflects, and is consistent with, the high levels of genetic diversity found in *B. carambolae* strains in studies of microsatellites and the COI gene, that appear to be related to their status as a native or invasive strain^16,17^. Nevertheless, we recognize that the limited number of strains assessed in this study requires further analysis of *pBac*^*Bd*-Kah^, and possibly other mutated transposons such as *hopper*^*Bd*-Kah^, ^46–48^ in a greater number of additional geographically dispersed strains for a clearer resolution of the relationship between *B. dorsalis* and *B. carambolae*.

The similar use of a transposon, or other mobile element-based genetic marker for common species identity should also be applicable to other eukaryotic organisms. These elements are ubiquitous throughout plants and animals, and for many organisms occupy a major portion of the genome. While the mobility of some insect transposons, such as the *P* element, are restricted to defined *Drosophila* species^49^, most currently identified transposons belong to less restricted superfamilies that have spread widely, presumably by horizontal transmission mediated by an infectious agent^50^. This may, indeed, be essential to the transposon lifecycle where it has been theorized that after horizontal transfer of a functional autonomous element, transposon replication and expansion occurs within a host genome with subsequent mutations rendering most genomic elements defective prior to their having a negative effect on viability^50^. However, once immobilized, random mutations accumulate in the non-autonomous elements and their descendants providing little or no selective pressure on their host, but act as a molecular clock that can be used to assess species identity or divergence. Such arrays of, primarily, defective mobile elements (that could occur in the hundreds to thousands) within a genome have previously been used as a retrotransposon signature marker for plant species identity based upon conserved restriction sites or PCR fragment lengths^26,27^. Insertional polymorphisms of non-autonomous MITE families have been used in barley^29^ and wheat^51^ as markers for specific genetic relationships as well as diversity, and copy number and genomic positions of active *gypsy* retrotransposons have been used in six wild coffee species to infer species boundaries and evolutionary history^52^. For the study presented here we not only use the common sequence and genomic position of an individual variant Class II transposon sequence to imply species identity, but its sequence divergence from the known functional IFP2 element has allowed an estimation of the length of time *pBac*^*Bd*-Kah^ homolog elements evolved in these strains, and the minimum length of time the taxa have remained conspecific^41,42,44^. This was calculated to be approximately 0.4 Myr for both the synonymized taxa and the *B. carambolae* strains (Table S2). This coincides fairly accurately with the minimum divergence times of 0.5 to 0.75 Myr calculated for other degenerate internal *piggyBac* sequences in the *B. dorsalis*, *B. zonata* and *B. tryoni* species complexes reported previously^25^. Thus, it is likely that these elements radiated from the same functional *piggyBac* invasion into an ancestral *Bactrocera* species.

Given the existence of numerous defective mobile elements in most organisms, the same phylogenetic relationships could be similarly defined, or at least improved upon, for species where ambiguities in relatedness exist. As demonstrated for the taxa closely related to *B. dorsalis s.s.*, eventual synonymization relied on the accumulation of independent consensus genetic data^7,9,10^, for which transposon-based markers can be an important component as demonstrated here, as well as in a variety of other organisms. This is especially important for insect pests where defining species identity has significant economic implications for methods of control and the implementation of trade barriers^9,21^. Indeed, identities in degenerate *piggyBac* elements within the lepidopterans, *Helicoverpa armigera* and *H. zea*, considered to be allopatric, have provided support for the notion that they are conspecific, although a common genomic insertion site has yet to be investigated^53^. Importantly, while numerous defective elements may be discovered in a species that can be related to a superfamily of elements, it is most useful to relate these elements to a specific functional ancestral element. In addition to *piggyBac*, for insects this may be provided by the *hAT* elements *Hermes* and *hopper*, the *Tc/mariner* elements *Mos1* and *Minos*, the *P* element specifically for *Drosophila* species, in addition to several families of retrotransposons^54,55^.

The need to test additional mobile element markers may also be required when results from a single marker are ambiguous, such as the absence of a homologous element or its presence in a distant insertion site, in strains otherwise known to be conspecific. For markers such as *pBac*^*Bd*-Kah^ that do not retain autonomous mobility due to a mutated transposase, such elements can be cross-mobilized by exogenous transposase if they retain intact ITR and sub-ITR sequences (see Fig. S1 for *pBac*^*Bd*-Kah^ ITRs). Thus, the *pBac*^*Bd*-Kah^ marker could be re-mobilized in specific individuals due to the unique presence of an invasive functional *piggyBac* or a closely related TTAA-element^56,57^, resulting in either loss of the marker or its genomic transposition. Indeed, the IFP2 element was first discovered in the genome of a *T. ni* cell line after it transposed into an infectious baculovirus^22^, and a similar baculovirus could have been the original source of the horizontally transmitted IFP2^50^.

In summary, we have demonstrated the applicability of using a nucleotide sequence comparison of a mutated non-mobile *piggyBac* transposable element and its genomic insertion site as a marker for common species identity in economically important invasive pest insects. These 3.2 kb sequences, having less than 95% nucleotide identity to the functional element, maintained greater than 99.5% identity among *B. dorsalis s.s* and three species previously synonymized with *B. dorsalis*. Strains of the closely related *B. carambolae* species, considered to be monophyletic, exhibited divergence from the *B. dorsalis* strains, but a limited sample size prevented a conclusive assessment of their relationship. Indeed, while we anticipate that the mutated descendants of *piggyBac* and other mobile genetic elements may be effective markers for conspecific identity in a wide range of organisms, the significance of these studies, and especially those aimed at assessing potential heterospecificity, should examine individuals from a broad range of strains that are geographically distinct, and where possible, tested with multiple mobile element markers.

### Supplementary Information

Supplementary Information.

## Data Availability

Nucleotide sequences that support the findings of this study can be viewed by the accession numbers provided in Table 1 (OQ718442 to OQ718454), at NCBI GenBank: https://www.ncbi.nlm.nih.gov/nuccore/. Other data will be made available upon request to the corresponding author.
