# Supplementary material for: A transposon-based genetic marker for conspecific identity within the Bactrocera dorsalis species complex
Source: Sci Rep. 2024 Jan 22;14:1924. doi: 10.1038/s41598-023-51068-2 (PMC10803768; doi:10.1038/s41598-023-51068-2)
Supplement: Supplementary file 1 — Supplementary Information. [file 41598_2023_51068_MOESM1_ESM.pdf]

Scientific Reports

Supplementary Information for:

A transposon-based genetic marker for conspecific identity within the *Bactrocera dorsalis* species complex

Grazyna J. Zimowska[1,2], Nirmala Xavier[1], Masroor Qadri[1] and Alfred M. Handler[1*]

[1]Center for Medical, Agricultural, and Veterinary Entomology, Agricultural Research Service, U.S. Department of Agriculture, 1700 SW 23rd Drive, Gainesville, FL, 32608 USA

[2]Entomology and Nematology Department, University of Florida, Gainesville, FL 32611

***Correspondence:** al.handler@usda.gov

Table of Contents:

Table S1
Table S2
Figure S1
Figure S2

Table S1. Oligonucleotide PCR primer sequences in 5’ to 3’ orientation

Primer	5’ nt binding site*	5’- 3’ sequence
AH147F	BdK5’gen -503	GATAGTTGTCCTTTACTTAAGCG
AH513F	BdK5’gen -427bp	CGCTTTGGAAGAGTTGGAGAC
AH153F	BdK5’gen -347bp	TGATTCGGACAGCAC
AH78R	IFP2_1297R	ACTCCGTTGGTCTGTGTTCC
AH143R	IFP2_169	ATAGTTCAAAATCAGTGACACTTACCG
Bd994R	BdK_387	TCGCTTTGCAGAAGAGCAGA
AH106F	BdK_599	GCGTAGCCGAGTCTCTG
AH108R	BdK_669	TATATTGCGGCACATACG
Bd1907R	BdK_1300	TAAAGGCGGAGTGGACA
AH107F	BdK_1600	TAAAGGCGGAGTGGACA
Bd1589F	BdK_981	CCCACACTTCGAGGAAACGA
AH144F	BdK_2332	CCTCGATATACAGACCGATAAAACAC
AH148R	IFP2_3212	GCATATCCATTGCCATCACC
Bd3052R	BdK3’gen +25	CAGCTCGAGAAAGCCAGGAA
Bd3229R	BdK3’gen +202	CGGTGGAATTTGTTGCTGCA
AH514R	BdK3’gen +371	ATGAGAGCCGTACTGTTGCC

*Binding site positions provided for either the *pBac*$^{Bd\text{-Kah}}$ (BdK) or the *T. ni* IFP2 *piggyBac* element (IFP2), although most internal primer sites are present in both elements . Positions designated as (-) and (+) are relative to the 5’ and 3’ *piggyBac* terminal nucleotides (nt), respectively.

Table S2. Divergence Times (in generations and years) and Confidence Intervals (CI) between the functional *T. ni* IFP2 *piggyBac* transposable element sequence and *piggyBac*$^{Bd\text{-}Kah}$ homologous sequences from indicated *Bactrocera* species and strains. The *Bactrocera g*eneration time is approximately 30 days (parental embryo to progeny embryo) resulting in ~12 generations per year, thus Divergence Times in Myr = # generations /12.

	Divergence Times (millions of generations)	95% CI lower	95% CI upper	Divergence Times (Myr)	95% CI lower	95% CI upper
Bcarambolae_Bangladesh	4.858569207	2.151122245	13.41890543	0.399334455	0.176804568	1.102923734
Bcarambolae_French_Guiana	4.85815118	2.150937164	13.41775088	0.399300097	0.176789356	1.102828839
Bcarambolae_Malaysia	4.858569207	2.151122245	13.41890543	0.399334455	0.176804568	1.102923734
Bcarambolae_Suriname	4.857811704	2.150786861	13.41681328	0.399272195	0.176777002	1.102751776
Bdorsalis_Kahuku	4.857811704	2.150786861	13.41681328	0.399272195	0.176777002	1.102751776
Bdorsalis_Hawaii-GSS	4.933136531	2.184136785	13.62485328	0.405463277	0.179518092	1.119850954
Binvadens_Kenya	4.857811704	2.150786861	13.41681328	0.399272195	0.176777002	1.102751776
Binvadens_Tanzania-DES2	5.083243415	2.250596321	14.03943419	0.417800829	0.18498052	1.153926098
Binvadens_Tanzania-KA	5.161696043	2.285331072	14.25611288	0.42424899	0.187835431	1.171735305
Bpapayae_Australia	5.007031231	2.216853522	13.8289434	0.411536813	0.182207139	1.136625485
Bpapayae_Malaysia	4.933136531	2.184136785	13.62485328	0.405463277	0.179518092	1.119850954
Bphilippinensis_Guimaras	4.857811704	2.150786861	13.41681328	0.399272195	0.176777002	1.102751776
Bphilippinensis_wild1	4.857811704	2.150786861	13.41681328	0.399272195	0.176777002	1.102751776

```
                               1        10        20        30        40        50        60        70        80
Tni IFP2_piggyBac              CCCTAGAAAGATAGTCTGCGTAAAATTGACGCATGCATTTCTTGAAATATTGCTCTCTCTTTCTAAATAGCGCGAATTCCGTCGCT
Bcarambolae_Bangladesh         CCCTAGAAAGATAGTCTGCGTAAAATTGACGCATGCATTTTTCGAAATATTGCTCTCTCTTTCTAAATAGCGCGAATTCCGTCGCT
Bcarambolae_French Guiana      CCCTAGAAAGATAGTCTGCGTAAAATTGACGCATGCATTTTTCGAAATATTGCTCTCTCTTTCTAAATAGCGCGAATTCCGTCGCT
Bcarambolae_Malaysia           CCCTAGAAAGATAGTCTGCGTAAAATTGACGCATGCATTTTTCGAAATATTGCTCTCTCTTTCTAAATAGCGCGAATTCCGTCGCT
Bcarambolae_Suriname           CCCTAGAAAGATAGTCTGCGTAAAATTGACGCATGCATTTTTCGAAATATTGCTCTCTCTTTCTAAATAGCGCGAATTCCGTCGCT
Bdorsalis_Kahuku               CCCTAGAAAGATAGTCTGCGTAAAATTGACGCATGCATTTTTCGAAATATTGCTCTCTCTTTCTAAATAGCGCGAATTCCGTCGCT
Bdorsalis_Hawaii-GSS           CCCTAGAAAGATAGTCTGCGTAAAATTGACGCATGCATTTTTCGAAATATTGCTCTCTCTTTCTAAATAGCGCGAATTCCGTCGCT
Binvadens_Kenya                CCCTAGAAAGATAGTCTGCGTAAAATTGACGCATGCATTTTTCGAAATATTGCTCTCTCTTTCTAAATAGCGCGAATTCCGTCGCT
Binvadens_Tanzania-DES2        CCCTAGAAAGATAGTCTGCGTAAAATTGACGCATGCATTTTTCGAAATATTGCTCTCTCTTTCTAAATAGCGCGAATTCCGTCGCT
Binvadens_Tanzania-KA          CCCTAGAAAGATAGTCTGCGTAAAATTGACGCATGCATTTTTCGAAATACTGCTCTCTCTTTCTAAATAGCGCGAACCCGTCGCT
Bpapayae_Australia             CCCTAGAAAGATAGTCTGCGTAAAATTGACGCATGCATTTTTCGAAATATTGCTCTCTCTTTCTAAATAGCGCGAATTCCGTCGCT
Bpapayae_Malaysia              CCCTAGAAAGATAGTCTGCGTAAAATTGACGCATGCATTTTTCGAAATATTGCTCTCTCTTTCTAAATAGCGCGAATTCCGTCGCT
Bphilippinensis_Guimaras       CCCTAGAAAGATAGTCTGCGTAAAATTGACGCATGCATTTTTCGAAATATTGCTCTCTCTTTCTAAATAGCGCGAATTCCGTCGCT
Bphilippinensis_wild1          CCCTAGAAAGATAGTCTGCGTAAAATTGACGCATGCATTTTTCGAAATATTGCTCTCTCTTTCTAAATAGCGCGAATTCCGTCGCT
```

```
                                   90       100       110       120       130       140       150       160
Tni IFP2_piggyBac              GTGCATTTAGGACATCTCAGTCGCC-GCTTGGAGCTCCCGTGAGGCGTGCTTGTCAATGCGGTAAGTGTCACTGATTTTGAACT
Bcarambolae_Bangladesh         GTGCGTTTAGGACATCTCAGTCGCC-GCTTGGAGCTCCCGTGAGGCGTGCTTGTCAATGCGGTAAGTGTCACTGATTTTGAAAT
Bcarambolae_French Guiana      GTGCGTTTAGGACATCTCAGTCGCC-GCTTGGAGCTCCCGTGAGGCGTGCTTGTCAATGCGGTAAGTGTCACTGATTTTGAAAT
Bcarambolae_Malaysia           GTGCGTTTAGGACATCTCAGTCGCC-GCTTGGAGCTCCCGTGAGGCGTGCTTGTCAATGCGGTAAGTGTCACTGATTTTGAAAT
Bcarambolae_Suriname           GTGCGTTTAGGACATCTCAGTCGCCNGCTTGGAGCTCCCGTGAGGCGTGCTTGTCAATGCGGTAAGTGTCACTGATTTTGAAAT
Bdorsalis_Kahuku               GTGCGTTTAGGACATCTCAGTCGCCTGCTTGGAGCTCCCGTGAGGCGTGCTTGTCAATGCGGTAAGTGTCACTGATTTTGAAAT
Bdorsalis_Hawaii-GSS           GTGCGTTTAGGACATCTCAGTCGCC-GCTTGGAGCTCCCGTGAGGCGTGCTTGTCAATGCGGTAAGTGTCACTGATTTTGAAAT
Binvadens_Kenya                GTGCGTTTAGGACATCTCAGTCGCC-GCTTGGAGCTCCCGTGAGGCGTGCTTGTCAATGCGGTAAGTGTCACTGATTTTGAAAT
Binvadens_Tanzania-DES2        GTGCGTTTAGGACATCTCAGTCGCC-GCTTGGAGCTCCCGTGAGGCGTGCTTGTCAATGCGGTAAGTGTCACTGATTTTGAAAT
Binvadens_Tanzania-KA          GTGCGTTTAGGACATCTCAGTCGCC-GCTTGGAGCTCCCGTGAGGCGTGCTTGTCAATGCGGTAAGTGTCACTGATTTTGAAAT
Bpapayae_Australia             GTGCGTTTAGGACATCTCAGTCGCC-GCTTGGAGCTCCCGTGAGGCGTGCTTGTCAATGCGGTAAGTGTCACTGATTTTGAAAT
Bpapayae_Malaysia              GTGCGTTTAGGACATCTCAGTCGCCNGCTTGGAGCTCCCGTGAGGCGTGCTTGTCAATGCGGTAAGTGTCACTGATTTTGAAAT
Bphilippinensis_Guimaras       GTGCGTTTAGGACATCTCAGTCGCC-GCTTGGAGCTCCCGTGAGGCGTGCTTGTCAATGCGGTAAGTGTCACTGATTTTGAAAT
Bphilippinensis_wild1          GTGCGTTTAGGACATCTCAGTCGCCNGCTTGGAGCTCCCGTGAGGCGTGCTTGTCAATGCGGTAAGTGTCACTGATTTTGAAAT
```

```
                               170       180       190       200       210       220       230       240       250
Tni IFP2_piggyBac              ATAACGACCGCGTGAGTCAAAATGACGCATGATTATCTTTTACGTGACTTTTAAGAT-TTAACTCATACGATAATTATATTGTT
Bcarambolae_Bangladesh         ATAACGACCGCGTGAGTCAAAATGACGCATGACTATCTTTTACGTATCTTTTAAGATTTTAACTTATACGATAATTATATTGTT
Bcarambolae_French Guiana      ATAACGACCGCGTGAGTCAAAATGACGCATGACTATCTTTTACGTATCTTTTAAGATTTTAACTTATACGATAATTATATTGTT
Bcarambolae_Malaysia           ATAACGACCGCGTGAGTCAAAATGACGCATGACTATCTTTTACGTATCTTTTAAGATTTTAACTTATACGATAATTATATTGTT
Bcarambolae_Suriname           ATAACGACCGCGTGAGTCGAAATGACGCATGACTATCTTTTACGTATCTTTTAAGATTTTAACTTATRCGATAATTATATTGTT
Bdorsalis_Kahuku               ATAACGACCGCGTGAGTCGAAATGACGCATGACTATCTTTTACGTATCTTTTAAGATTTTAACTTATACGATAATTATATTGTT
Bdorsalis_Hawaii-GSS           ATAACGACCGCGTGAGTCGAAATGACGCATGACTATCTTTTACGTATCTTTTAAGATTTTAACTTATACGATAATTATATTGTT
Binvadens_Kenya                ATAACGACCGCGTGAGTCGAAATGACGCATGACTATCTTTTACGTATCTTTTAAGATTTTAACTTATACGATAATTATATTGTT
Binvadens_Tanzania-DES2        ATAACGACCGCGTGAGTCGAAATGACGCATGACTATCTTTTACGTATCTTTTAAGATTTTAACTTATACGATAATTATATTGTT
Binvadens_Tanzania-KA          ATAACGACCGCGTGAGTCGAAATGACGCATGACTATCTTTTACGTATCTTTTAAGATTTTAACTTATACGATAATTATATTGTT
Bpapayae_Australia             ATAACGACCGCGTGAGTCGAAATGACGCATGACTATCTTTTACGTATCTTTTAAGATTTTAACTTATACGATAATTATATTGTT
Bpapayae_Malaysia              ATAACGACCGCGTGAGTCGAAATGACGCATGACTATCTTTTACGTATCTTTTAAGATTTTAACTTATACGATAATTATATTGTT
Bphilippinensis_Guimaras       ATAACGACCGCGTGAGTCGAAATGACGCATGACTATCTTTTACGTATCTTTTAAGATTTTAACTTATACGATAATTATATTGTT
Bphilippinensis_wild1          ATAACGACCGCGTGAGTCGAAATGACGCATGACTATCTTTTACGTATCTTTTAAGATTTTAACTTATACGATAATTATATTGTT
```

```
                                       260       270       280       290       300       310       320       330
Tni IFP2_piggyBac              ATTTCATGTTCTACTTACGTGATAACTTATTATATATA----TATTTTCTTGTTATAGATATCGTGACTAATATATAATAAAAT
Bcarambolae_Bangladesh         ATTTCATGTTCTACTTACGTGATAACTTATTATATATATATTTGTTTTCTTGTTATAGATATCGTGACTAAAATATAATAAAAT
Bcarambolae_French Guiana      ATTTCATGTTCTACTTACGTGATAACTTATTATATATATATTTGTTTTCTTGTTATAGATATCGTGACTAAAATATAATAAAAT
Bcarambolae_Malaysia           ATTTCATGTTCTACTTACGTGATAACTTATTATATATATATTTGTTTTCTTGTTATAGATATCGTGACTAAAATATAATAAAAT
Bcarambolae_Suriname           ATTTCATGTTCTACTTACGTGATAACTTATTATATATATATTTGTTTTCTTGTTATAGATATCGTGACTAAAATATAATAAAAT
Bdorsalis_Kahuku               ATTTCATGTTCTACTTACGTGATAACTTATTATATATATATTTGTTTTCTTGTTATAGATATCGTGACTAAAATATAATAAAAT
Bdorsalis_Hawaii-GSS           ATTTCATGTTCTACTTACGTGATAACTTATTATATATATATTTGTTTTCTTGTTATAGATATCGTGACTAAAATATAATAAAAT
Binvadens_Kenya                ATTTCATGTTCTACTTACGTGATAACTTATTATATATATATTTGTTTTCTTGTTATAGATATCGTGACTAAAATATAATAAAAT
Binvadens_Tanzania-DES2        ATTTCATGTTCTACTTACGTGATAACTTATTATATATATATTTGTTTTCTTGTTATAGATATCGTGACTAAAATATTATAAAAT
Binvadens_Tanzania-KA          ATTTCATGTTCTACTTACGTGATAACTTATTATATATATATTTGTTTTCTTGTTATAGATATCGTGACTAAAATATAATAAAAT
Bpapayae_Australia             ATTTCATGTTCTACTTACGTGATAACTTATTATATATATATTTGTTTTCTTGTTATAGATATCGTGACTAAAATATAATAAAAT
Bpapayae_Malaysia              ATTTCATGTTCTACTTACGTGATAACTTATTATATATATATTTGTTTTCTTGTTATAGATATCGTGACTAAAATATAATAAAAT
Bphilippinensis_Guimaras       ATTTCATGTTCTACTTACGTGATAACTTATTATATATATATTTGTTTTCTTGTTATAGATATCGTGACTAAAATATAATAAAAT
Bphilippinensis_wild1          ATTTCATGTTCTACTTACGTGATAACTTATTATATATATATTTGTTTTCTTGTTATAGATATCGTGACTAAAATATAATAAAAT
```

```
                                  340       350       360       370       380       390       400       410       420
Tni IFP2_piggyBac              GGGTAGTTCTTTAGACGATGAGCAATATCCTCTCTGCTCTTCTGCAAAGCGATGACGAGCTTGTTGGTGAGGATTCTGACAGTGA
Bcarambolae_Bangladesh         GGGTAGTCCTTTAGACAATGAGCGTATCCTCTCTGCTCTTATGCAAAGCGATGACGAGCTCGTTGGTGAGGATTCTGACAGTGA
Bcarambolae_French Guiana      GGGTAGTCCTTTAGACAATGAGCGTATCCTCTCTGCTCTTCTGCAAAGCGATGACGAGCTCGTTGGTGAGGATTCTGACAGTGA
Bcarambolae_Malaysia           GGGTAGTCCTTTAGACAATGAGCGTATCCTCTCTGCTCTTATGCAAAGCGATGACGAGCTCGTTGGTGAGGATTCTGACAGTGA
Bcarambolae_Suriname           GGGTAGTCCTTTAGACAATGAGCGTATCCTCTCTGCTCTTCTGCAAAGCGATGACGAGCTCGTTGGTGAGGATTCTGACAGTGA
Bdorsalis_Kahuku               GGGTAGTCCTTTAGACAATGAGCGTATCCTCTCTGCTCTTCTGCAAAGCGATGACGAGCTCGTTGGTGAGGATTCTGACAGTGA
Bdorsalis_Hawaii-GSS           GGGTAGTCCTTTAGACAATGAGCGTATCCTCTCTGCTCTTCTGCAAAGCGATGACGAGCTCGTTGGTGAGGATTCTGACAGTGA
Binvadens_Kenya                GGGTAGTCCTTTAGACAATGAGCGTATCCTCTCTGCTCTTCTGCAAAGCGATGACGAGCTCGTTGGTGAGGATTCTGACAGTGA
Binvadens_Tanzania-DES2        GGGTAGTCCTTTAGACAATGAGCGTATCCTCTCTGCTCTTCTGCAAAGCGATGACGAGCTCGTTGGTGAGGATTCTGACAGTGA
Binvadens_Tanzania-KA          GGGTAGTCCTTTAGACAATGAGCGTATCCTCTCTGCTCTTCTGCAAAGCGATGACGAGCTCGTTGGTGAGGATTCTGACAGTGA
Bpapayae_Australia             GGGTAGTCCTTTAGACAATGAGCGTATCCTCTCTGCTCTTCTGCAAAGCGATGACGAGCTCGTTGGTGAGGATTCTGACAGTGA
Bpapayae_Malaysia              GGGTAGTCCTTTAGACAATGAGCGTATCCTCTCTGCTCTTCTGCAAAGCGATGACGAGCTCGTTGGTGAGGATTCTGACAGTGA
Bphilippinensis_Guimaras       GGGTAGTCCTTTAGACAATGAGCGTATCCTCTCTGCTCTTCTGCAAAGCGATGACGAGCTCGTTGGTGAGGATTCTGACAGTGA
Bphilippinensis_wild1          GGGTAGTCCTTTAGACAATGAGCGTATCCTCTCTGCTCTTCTGCAAAGCGATGACGAGCTCGTTGGTGAGGATTCTGACAGTGA
```

```
                               430       440       450       460       470       480       490       500
Tni IFP2_piggyBac              AATATCAGATCACGTAAGTGAAGATGACGTCCAGAGCGATACAGAAGAAGCGTTTATAGATGAGGTACATGAAGTGCAGCCAAC
Bcarambolae_Bangladesh         AATATCAGATCACGTAAGTGAAGATGACGTCCAGAGCGATACAGAAGAAGCGTTTATAGATGAGGTGCATGAAGTGCAGCCAAC
Bcarambolae_French Guiana      AATATCAGATCACGTAAGTGAAGATGACGTCCAGAGCGATACAGAAGAAGCGTTTATAGATGAGGTGCATGAAGTGCAGCCAAC
Bcarambolae_Malaysia           AATATCAGATCACGTAAGTGAAGATGACGTCCAGAGCGATACAGAAGAAGCGTTTATAGATGAGGTGCATGAAGTGCAGCCAAC
Bcarambolae_Suriname           AATATCAGATCACGTAAGTGAAGATGACGTCCAGAGCGATACAGAAGAAG----------------------------------
Bdorsalis_Kahuku               AATATCAGATCACGTAAGTGAAGATGACGTCCAGAGCGATACAGAAGAAG----------------------------------
Bdorsalis_Hawaii-GSS           AATATCAGATCACGTAAGTGAAGATGACGTCCAGAGCGATACAGAAGAAG----------------------------------
Binvadens_Kenya                AATATCAGATCACGTAAGTGAAGATGACGTCCAGAGCGATACAGAAGAAG----------------------------------
Binvadens_Tanzania-DES2        AATATCAGATCACGTAAGTGAAGGTGACGTCCAGAGCGATACAGAAGAAG----------------------------------
Binvadens_Tanzania-KA          AATATCAGATCACGTAAGTGAAGATGACGTCCAGAGCGATACAGAAGAAG----------------------------------
Bpapayae_Australia             AATATCAGATCACGTAAGTGAAGATGACGTCCAGAGCGATACAGAAGAAG----------------------------------
Bpapayae_Malaysia              AATATCAGATCACGTAAGTGAAGATGACGTCCAGAGCGATACAGAAGAAG----------------------------------
Bphilippinensis_Guimaras       AATATCAGATCACGTAAGTGAAGATGACGTCCAGAGCGATACAGAAGAAG----------------------------------
Bphilippinensis_wild1          AATATCAGATCACGTAAGTGAAGATGACGTCCAGAGCGATACAGAAGAAG----------------------------------

                               510       520       530       540       550       560       570       580
Tni IFP2_piggyBac              GTCAAGCGGTAGTGAAATATTAGACGAACAAAATGTTATTGAACAACCAGGTTCTTCATTGGCTTCTAACAGAATCTTGACCTT
Bcarambolae_Bangladesh         GTCAAGCGGTAGTGAAATATTAGA---ACAAAATATTATTGAACAACCAGGTTCTTCATTGGCTTCTAACAGAATCTTGACCTT
Bcarambolae_French Guiana      GTCAAGCGGTAGTGAAATATTAGA---ACAAAATATTATTGAACAACCAGGTTCTTCATTGGCTTCTAACAGAATCTTGACCTT
Bcarambolae_Malaysia           GTCAAGCGGTAGTGAAATATTAGA---ACAAAATATTATTGAACAACCAGGTTCTTCATTGGCTTCTAACAGAATCTTGACCTT
Bcarambolae_Suriname           ------------CGAAATATTAGA---ACAAAATATTATTGAACAACCAGGTTCTTCATTGGCTTCTAACAGAATCTTGACCTT
Bdorsalis_Kahuku               ------------CGAAATATTAGA---ACAAAATATTATTGAACAACCAGGTTCTTCATTGGCTTCTAACAGAATCTTGACCTT
Bdorsalis_Hawaii-GSS           ------------CGAAATATTAGA---ACAAAATATTATTGAACAACCAGGTTCTTCATTGGCTTCTAACAGAATCTTGACCTT
Binvadens_Kenya                ------------CGAAATATTAGA---ACAAAATATTATTGAACAACCAGGTTCTTCATTGGCTTCTAACAGAATCTTGACCTT
Binvadens_Tanzania-DES2        ------------CGAAATATTAGA---ACAAAATATTATTGAACAACCAGGTTCTTCATTGGCTTCTAACAGAATCTTGACCTT
Binvadens_Tanzania-KA          ------------CGAAATATTAGA---ACAAAATATTATTGAACAACCAGGTTCTTCATTGGCTTCTAACAGAATCTTGACCTT
Bpapayae_Australia             ------------CGAAATATTAGA---ACAAAATATTATTGAACAACCAGGTTCTTCATTGGCTTCTAACAGAATCTTGACCTT
Bpapayae_Malaysia              ------------CGAAATATTAGA---ACAAAATATTATTGAACAACCAGGTTCTTCATTGGCTTCTAACAGAATCTTGACCTT
Bphilippinensis_Guimaras       ------------CGAAATATTAGA---ACAAAATATTATTGAACAACCAGGTTCTTCATTGGCTTCTAACAGAATCTTGACCTT
Bphilippinensis_wild1          ------------CGAAATATTAGA---ACAAAATATTATTGAACAACCAGGTTCTTCATTGGCTTCTAACAGAATCTTGACCTT

                               590       600       610       620       630       640       650       660       670
Tni IFP2_piggyBac              GCCACAGAGGACTATTAGAGGTAAGAATAAACATTGTTGGTCAACTTCAAAGTCCACGAGGCGTAGCCGAGTCTCTGCACTGAA
Bcarambolae_Bangladesh         GCCACAGAGGACTATTAGAGGTAAGAATAAACATTGTTGGTCAACTTCAAAGTCCACGAGGCGTAGCCGAGTCTCCGCACTGAA
Bcarambolae_French Guiana      GCCACAGAGGACTATTAGAGGTAAGAATAAACATTGTTGGTCAACTTCAAAGTCCACGAGGCGTAGCCGAGTCTCTGCACTGAA
Bcarambolae_Malaysia           GCCACAGAGGACTATTAGAGGTAAGAATAAACATTGTTGGTCAACTTCAAAGTCCACGAGGCGTAGCCGAGTCTCCGCACTGAA
Bcarambolae_Suriname           GCCACAGAGGACTATTAGAGGTAAGAATAAACATTGTTGGTCAACTTCAAAGTCCACGAGGCGTAGCCGAGTCTCTGCACTGAA
Bdorsalis_Kahuku               GCCACAGAGGACTATTAGAGGTAAGAATAAACATTGTTGGTCAACTTCAAAGTCCACGAGGCGTAGCCGAGTCTCTGCACTGAA
Bdorsalis_Hawaii-GSS           GCCACAGAGGACTATTAGAGGTAAGAATAAACATTGTTGGTCAACTTCAAAGTCCACGAGGCGTAGCCGAGTCTCTGCACTGAA
Binvadens_Kenya                GCCACAGAGGACTATTAGAGGTAAGAATAAACATTGTTGGTCAACTTCAAAGTCCACGAGGCGTAGCCGAGTCTCTGCACTGAA
Binvadens_Tanzania-DES2        GCCACAGAGGACTATTAGAGGTAAGAATAAACATTGTTGGTCAACTTCAAAGTCCACGAGGCGTAGCCGAGTCTCTGCACTGAA
Binvadens_Tanzania-KA          GCCACAGAGGACTATTAGAGGTAAGAATAAACATTGTTGGTCAACTTCAAAGTCCACGAGGCGTAGCCGAGTCTCTGCACTGAA
Bpapayae_Australia             GCCACAGAGGACTATTAGAGGTAAGAATAAACATTGTTGGTCAACTTCAAAGTCCACGAGGCGTAGCCGAGTCTCTGCACTGAA
Bpapayae_Malaysia              GCCACAGAGGACTATTAGAGGTAAGAATAAACATTGTTGGTCAACTTCAAAGTCCACGAGGCGTAGCCGAGTCTCTGCACTGAA
Bphilippinensis_Guimaras       GCCACAGAGGACTATTAGAGGTAAGAATAAACATTGTTGGTCAACTTCAAAGTCCACGAGGCGTAGCCGAGTCTCTGCACTGAA
Bphilippinensis_wild1          GCCACAGAGGACTATTAGAGGTAAGAATAAACATTGTTGGTCAACTTCAAAGTCCACGAGGCGTAGCCGAGTCTCTGCACTGAA

                               680       690       700       710       720       730       740       750
Tni IFP2_piggyBac              CATTGTCAGATCTCAAAGAGGTCCGACGCGTATGTGCCGCAATATATATGACCCACTTTTATGCTTCAAACTATTTTTTACTGA
Bcarambolae_Bangladesh         CATTGTCAGATCTCAAAGAGGTCCGACGCGTATGTGCCGCAATATATATGACCCACTTTTATGCTTCAAACTATTTTTTACTGA
Bcarambolae_French Guiana      CATTGTCAGATCTCAAAGAGGTCCGACGCGTATGTGCCGCAATATATATGACCCACTTTTATGCTTCAAACTATTTTTTACTGA
Bcarambolae_Malaysia           CATTGTCAGATCTCAAAGAGGTCCGACGCGTATGTGCCGCAATATATATGACCCACTTTTATGCTTCAAACTATTTTTTACTGA
Bcarambolae_Suriname           CATTGTCAGATCTCAAAGAGGTCCGACGCGTATGTGCCGCAATATATATGACCCACTTTTATGCTTCAAACTATTTTTTACTGA
Bdorsalis_Kahuku               CATTGTCAGATCTCAAAGAGGTCCGACGCGTATGTGCCGCAATATATATGACCCACTTTTATGCTTCAAACTATTTTTTACTGA
Bdorsalis_Hawaii-GSS           CATTGTCAGATCTCAAAGAGGTCCGACGCGTATGTGCCGCAATATATATGACCCACTTTTATGCTTCAAACTATTTTTTACTGA
Binvadens_Kenya                CATTGTCAGATCTCAAAGAGGTCCGACGCGTATGTGCCGCAATATATATGACCCACTTTTATGCTTCAAACTATTTTTTACTGA
Binvadens_Tanzania-DES2        CATTGTCAGATCTCAAAGAGGTCCGACGCGTATGTGCCGCAATATATATGACCCACTTTTATGCTTCAAACTATTTTTTACTGA
Binvadens_Tanzania-KA          CATTGTCAGATCTCAAAGAGGTCCGACGCGTATGTGCCGCAATATATATGACCCACTTTTATGCTTCAAACTATTTTTTACTGA
Bpapayae_Australia             CATTGTCAGATCTCAAAGAGGTCCGACGCGTATGTGCCGCAATATATATGACCCACTTTTATGCTTCAAACTATTTTTTACTGA
Bpapayae_Malaysia              CATTGTCAGATCTCAAAGAGGTCCGACGCGTATGTGCCGCAATATATATGACCCACTTTTATGCTTCAAACTATTTTTTACTGA
Bphilippinensis_Guimaras       CATTGTCAGATCTCAAAGAGGTCCGACGCGTATGTGCCGCAATATATATGACCCACTTTTATGCTTCAAACTATTTTTTACTGA
Bphilippinensis_wild1          CATTGTCAGATCTCAAAGAGGTCCGACGCGTATGTGCCGCAATATATATGACCCACTTTTATGCTTCAAACTATTTTTTACTGA

                               760       770       780       790       800       810       820       830       840
Tni IFP2_piggyBac              TGAGATAATTTCGGAAATTGTAAAATGGACAAATGCTGAGATATCATTGAAACGTCGGGAATCTATGACAGGTGCTACATTTCG
Bcarambolae_Bangladesh         TGAGATAATTTCGGAAATCGTAAAATGGACAAATTCTGAGATATCATTGAAACGTCGGGAATCTATGACAGATGCTACATTTCG
Bcarambolae_French Guiana      TGAGATAATTTCGGAAATCGTAAAATGGACAAATTCTGAGATATCATTGAAACGTCGGTAATCTATGACAGATGCTACATTTCG
Bcarambolae_Malaysia           TGAGATAATTTCGGAAATCGTAAAATGGACAAATTCTGAGATATCATTGAAACGTCGGGAATCTATGACAGATGCTACATTTCG
Bcarambolae_Suriname           TGAGATAATTTCGGAAATCGTAAAATGGACAAATGCTGAGATATCATTGAAACGTCGGGAATCTATGACAGGTGCTACATTTCG
Bdorsalis_Kahuku               TGAGATAATTTCGGAAATCGTAAAATGGACAAATGCTGAGATATCATTGAAACGTCGGGAATCTATGACAGGTGCTACATTTCG
Bdorsalis_Hawaii-GSS           TGAGATAATTTCGGAAATCGTAAAATGGACAAATGCTGAGATATCATTGAAACGTCGGGAATCTATGACAGGTGCTACATTTCG
Binvadens_Kenya                TGAGATAATTTCGGAAATCGTAAAATGGACAAATGCTGAGATATCATTGAAACGTCGGGAATCTATGACAGGTGCTACATTTCG
Binvadens_Tanzania-DES2        TGAGATAATTTCGGAAATCGTAAAATGGACAAATGCTGAGATATCATTGAAACGTCGGGAATCTATGACAGGTGCTACATTTCG
Binvadens_Tanzania-KA          TGAGATAATTTCGGAAATCGTAAAATGGACAAATGCTGAGATATCATTGAAACGTCGGGAATCTATGACAGGTGCTACATTTCG
Bpapayae_Australia             TGAGATAATTTCGGAAATCGTAAAATGGACAAATGCTGAGATATCATTGAAACGTCGGGAATCTATGACAGGTGCTACATTTCG
Bpapayae_Malaysia              TGAGATAATTTCGGAAATCGTAAAATGGACAAATGCTGAGATATCATTGAAACGTCGGGAATCTATGACAGGTGCTACATTTCG
Bphilippinensis_Guimaras       TGAGATAATTTCGGAAATCGTAAAATGGACAAATGCTGAGATATCATTGAAACGTCGGGAATCTATGACAGGTGCTACATTTCG
Bphilippinensis_wild1          TGAGATAATTTCGGAAATCGTAAAATGGACAAATGCTGAGATATCATTGAAACGTCGGGAATCTATGACAGGTGCTACATTTCG
```

```
                              850       860       870       880       890       900       910       920
Tni IFP2_piggyBac             TGACACGAATGAAGATGAAATCTATGCTTTCTTTGGTATTCTGGTAATGACAGCAGTGAGAAAAGATAAYCACATGTCCACAGA
Bcarambolae_Bangladesh        TGACACAAATGAAGATGAAATCCATGCTTTGTTTGGTATTCTGGTAATGACAGCAGTGAGAAAAGATAACCACATGTCCACAGA
Bcarambolae_French Guiana     TGACACAAATGAAGATGAAATCCATGCTTTGTTTGGTATTCTGGTAATGACAGCAGTGAGAAAAGATAACCACATGTCCACAGA
Bcarambolae_Malaysia          TGACACAAATGAAGATGAAATCCATGCTTTGTTTGGTATTCTGGTAATGACAGCAGTGAGAAAAGATAACCACATGTCCACAGA
Bcarambolae_Suriname          AGACACGAATGAAGATGAAATCCATGCTTTGTTTGGTATTCTGGTAATGACAGCAGTGAGAAAAGATAACCACATGTCCACAGA
Bdorsalis_Kahuku              AGACACGAATGAAGATGAAATCCATGCTTTGTTTGGTATTCTGGTAATGACAGCAGTGAGAAAAGATAACCACATGTCCACAGA
Bdorsalis_Hawaii-GSS          AGACACGAATGAAGATGAAATCCATGCTTTGTTTGGTATTCTGGTAATGACAGCAGTGAGAAAAGATAACCACATGTCCACAGA
Binvadens_Kenya               AGACACGAATGAAGATGAAATCCATGCTTTGTTTGGTATTCTGGTAATGACAGCAGTGAGAAAAGATAACCACATGTCCACAGA
Binvadens_Tanzania-DES2       AGACACGAATGAAGATGAAATCCATGCTTTGTTTGGTATTCTGGTAATGACAGCAGTGAGAAAAGATAACCACATGTCCACAGA
Binvadens_Tanzania-KA         AGACACGAATGAAGATGAAATCCATGCTTTGTTTGGTATTCTGGTAATGACAGCAGTGAGAAAAGATAACCACATGTCCACAGA
Bpapayae_Australia            AGACACGAATGAAGATGAAATCCATGCTTTGTTTGGTATTCTGGTAATGACAGCAGTGAGAAAAGATAACCACATGTCCACAGA
Bpapayae_Malaysia             AGACACGAATGAAGATGAAATCCATGCTTTGTTTGGTATTCTGGTAATGACAGCAGTGAGAAAAGATAACCACATGTCCACAGA
Bphilippinensis_Guimaras      AGACACGAATGAAGATGAAATCCATGCTTTGTTTGGTATTCTGGTAATGACAGCAGTGAGAAAAGATAACCACATGTCCACAGA
Bphilippinensis_wild1         AGACACGAATGAAGATGAAATCCATGCTTTGTTTGGTATTCTGGTAATGACAGCAGTGAGAAAAGATAACCACATGTCCACAGA

                                 930       940       950       960       970       980       990       1,000
Tni IFP2_piggyBac             TGACCTCTTTGATCGATCTTTGTCAATGGTGTACGTCTCTGTAATGAGTCGTGATCGTTTTGATTTTTTGATACGATGTCTTAG
Bcarambolae_Bangladesh        TGACCTCTATGATCGATCTTTGTCAATGGTGTACGTCTCTGTAATGAGTCGTGATCGTTTTGATTTTTTGATACGATGTCTTAG
Bcarambolae_French Guiana     TGACCTCTATGATCGATCTTTGTCAATGGTGTACGTCTCTGTAATGAGTCGTGGTCGTTTTGATTTTTTGATACGATGTCTTAG
Bcarambolae_Malaysia          TGACCTCTATGATCGATCTTTGTCAATGGTGTACGTCTCTGTAATGAGTCGTGATCGTTTTGATTTTTTGATACGATGTCTTAG
Bcarambolae_Suriname          TGACCTCTATGATCGATCTTTGTCAATGGTGTACGTCTCTGTAATGAGTCGTGATCGTTTTGATTTTTTGATACGATGTCTTCG
Bdorsalis_Kahuku              TGACCTCTATGATCGATCTTTGTCAATGGTGTACGTCTCTGTAATGAGTCGTGATCGTTTTGATTTTTTGATACGATGTCTTCG
Bdorsalis_Hawaii-GSS          TGACCTCTATGATCGATCTTTGTCAATGGTGTACGTCTCTGTAATGAGTCGTGATCGTTTTGATTTTTTGATACGATGTCTTCG
Binvadens_Kenya               TGACCTCTATGATCGATCTTTGTCAATGGTGTACGTCTCTGTAATGAGTCGTGATCGTTTTGATTTTTTGATACGATGTCTTCG
Binvadens_Tanzania-DES2       TGACCTCTATGATCGATCTTTGTCAATGGTGTACGTCTCTGTAATGAGTCGTGATCGTTTTGATTTTTTGATACGATGTCTTCG
Binvadens_Tanzania-KA         TGACCTCTATGATCGACCTTTGTCAATGGTGTACGTCTCTGTAATGAGTCGTGATCGTTTTGATTTTTTGATACGATGTCTTCG
Bpapayae_Australia            TGACCTCTATGATCGATCTTTGTCAATGGTGTACGTCTCTGTAATGAGTCGTGATCGTTTTGATTTTTTGATACGATGTCTTCG
Bpapayae_Malaysia             TGACCTCTATGATCGATCTTTGTCAATGGTGTACGTCTCTGTAATGAGTCGTGATCGTTTTGATTTTTTGATACGATGTCTTCG
Bphilippinensis_Guimaras      TGACCTCTATGATCGATCTTTGTCAATGGTGTACGTCTCTGTAATGAGTCGTGATCGTTTTGATTTTTTGATACGATGTCTTCG
Bphilippinensis_wild1         TGACCTCTATGATCGATCTTTGTCAATGGTGTACGTCTCTGTAATGAGTCGTGATCGTTTTGATTTTTTGATACGATGTCTTCG

                              1,010     1,020     1,030     1,040     1,050     1,060     1,070     1,080     1,090
Tni IFP2_piggyBac             AATGGATGACAAAAGTATACGGCCCACACTTCGAGAAAACGATGTATTTACTCCTGTTAGAAAAATATGGGATCTCTTTATCCA
Bcarambolae_Bangladesh        AATGGATGACAAAAGTATACGGCCCACACTTCGAGGAAACGATGTATTTACTCCTGTTAGAAAAATATGGGATCTCTTTATTCA
Bcarambolae_French Guiana     AATGGATGACAAAAGTATACGGCCCACACTTCGAGGAAACGATGTATTTACTCCTGTTAGAAAAATATGGGATCTCTTTATTCA
Bcarambolae_Malaysia          AATGGATGACAAAAGTATACGGCCCACACTTCGAGGAAACGATGTATTTACTCCTGTTAGAAAAATATGGGATCTCTTTATTCA
Bcarambolae_Suriname          AATGGATGACAAAAGTATACGGCCCACACTTCGAGGAAACGATGTATTTACTCCTGTTAGAAAAATATGGGATCTCTTTATCCA
Bdorsalis_Kahuku              AATGGATGACAAAAGTATACGGCCCACACTTCGAGGAAACGATGTATTTACTCCTGTTAGAAAAATATGGGATCTCTTTATCCA
Bdorsalis_Hawaii-GSS          AATGGATGACAAAAGTATACGGCCCACACTTCGAGGAAACGATGTATTTACTCCTGTTAGAAAAATATGGGATCTCTTTATCCA
Binvadens_Kenya               AATGGATGACAAAAGTATACGGCCCACACTTCGAGGAAACGATGTATTTACTCCTGTTAGAAAAATATGGGATCTCTTTATCCA
Binvadens_Tanzania-DES2       AATGGATGACAAAAGTATACGGCCCACACTTCGAGGAAACGATGTATTTACTCCTGTTAGAAAAATATGGGATCTCTTTATCCA
Binvadens_Tanzania-KA         AATGGATGACAAAAGTATACGGCCCACACTTCGAGGAAACGATGTATTTACTCCTGTTAGAAAAATATGGGATCTCTTTATCCA
Bpapayae_Australia            AATGGATGACAAAAGTATACGGCCCACACTTCGAGGAAACGATGTATTTACTCCTGTTAGAAAAATATGGGATCTCTTTATCCA
Bpapayae_Malaysia             AATGGATGACAAAAGTATACGGCCCACACTTCGAGGAAACGATGTATTTACTCCTGTTAGAAAAATATGGGATCTCTTTATCCA
Bphilippinensis_Guimaras      AATGGATGACAAAAGTATACGGCCCACACTTCGAGGAAACGATGTATTTACTCCTGTTAGAAAAATATGGGATCTCTTTATCCA
Bphilippinensis_wild1         AATGGATGACAAAAGTATACGGCCCACACTTCGAGGAAACGATGTATTTACTCCTGTTAGAAAAATATGGGATCTCTTTATCCA

                                    1,100     1,110     1,120     1,130     1,140     1,150     1,160     1,170
Tni IFP2_piggyBac             TCAGTGCATACAAAATTACACTCCAGGGGCTCATTTGACCATAGATGAACAGTTACTTGGTTTTAGAGGACGGTGTCCGTTTAG
Bcarambolae_Bangladesh        TCAGTGCATACATAATTACACTCCAGGGGCTCATTTGACCATAGATGAACAGTTACTTGGGTTTAGAGGACGGTGTCCGTTTAG
Bcarambolae_French Guiana     TCAGTGCATACATAATTACACTCCAGGGGCTCATTTGACCATAGATGAACAGTTACTTGGGTTTAGAGGACGGTGTCCGTTTAG
Bcarambolae_Malaysia          TCAGTGCATACATAATTACACTCCAGGGGCTCATTTGACCATAGATGAACAGTTACTTGGGTTTAGAGGACGGTGTCCGTTTAG
Bcarambolae_Suriname          TCAGTGCATACATAATTACACTCCAGGGGCTCATTTGACCATAGATGAACAGTTACTTGGGTTTAGAGGACGGTGTCCGTTTAG
Bdorsalis_Kahuku              TCAGTGCATACATAATTACACTCCAGGGGCTCATTTGACCATAGATGAACAGTTACTTGGGTTTAGAGGACGGTGTCCGTTTAG
Bdorsalis_Hawaii-GSS          TCAGTGCGTACATAATTACACTCCAGGGGCTCATTTGACCATAGATGAACAGTTACTTGGGTTTAGAGGACGGTGTCCGTTTAG
Binvadens_Kenya               TCAGTGCATACATAATTACACTCCAGGGGCTCATTTGACCATAGATGAACAGTTACTTGGGTTTAGAGGACGGTGTCCGTTTAG
Binvadens_Tanzania-DES2       TCAGTGCATACATAATTACACTCCAGGGGCTCATTTGACCATAGATGAACAGTTACTTGGGTTTAGAGGACGGTGTCCGTTTAG
Binvadens_Tanzania-KA         TCAGTGCATACATAATTACACTCCAGGGGCTCATTTGACCATAGATGAACAGTTACTTGGGTTTAGAGGACGGTGTCCGTTTAG
Bpapayae_Australia            TCAGTGCATACATAATTACACTCCAGGGGCTCATTTGACCATAGATGAACAGTTACTTGGGTTTAGAGGACGGTGTCCGTTTAG
Bpapayae_Malaysia             TCAGTGCATACATAATTACACTCCAGGGGCTCATTTGACCATAGATGAACAGTTACTTGGGTTTAGAGGACGGTGTCCGTTTAG
Bphilippinensis_Guimaras      TCAGTGCATACATAATTACACTCCAGGGGCTCATTTGACCATAGATGAACAGTTACTTGGGTTTAGAGGACGGTGTCCGTTTAG
Bphilippinensis_wild1         TCAGTGCATACATAATTACACTCCAGGGGCTCATTTGACCATAGATGAACAGTTACTTGGGTTTAGAGGACGGTGTCCGTTTAG

                                1,180     1,190     1,200     1,210     1,220     1,230     1,240     1,250     1,260
Tni IFP2_piggyBac             GATGTATATCCCAAACAAGCCAAGTAAGTATGGAATAAAAATCCTCATGATGTGTGACAGTGGTACGAAGTATAT-GATAAATG
Bcarambolae_Bangladesh        AATGTATATTCCCAACAAGCCAAGTAAGTATGGAATAAAAATCCTCATGATGTGTGACAGTGGTACAAAGTACAT-GATAAATC
Bcarambolae_French Guiana     AATGTATATTCCCAACAAGCCAAGTAAGTATGGAATAAAAATCCTCATGATGTGTGACAGTGGTACAAAGTACAT-GATAAATC
Bcarambolae_Malaysia          AATGTATATTCCCAACAAGCCAAGTAAGTATGGAATAAAAATCCTCATGATGTGTGACAGTGGTACAAAGTACAT-GATAAATC
Bcarambolae_Suriname          AATGTATATTCCCAACAAGCCAAGTAAGTATGGAATAAAAATCCTCATGATGTGTGACAGTGGTACMAAGCACAT-GATAAATG
Bdorsalis_Kahuku              AATGTATATTCCCAACAAGCCAAGTAAGTATGGAATAAAAATCCTCATGATGTGTGACAGTGGTACCAAGCACAT-GATAAATG
Bdorsalis_Hawaii-GSS          AATGTATATTCCCAACAAGCCAAGTAAGTATGGAATAAAAATCCTCATGATGTGTGACAGTGGTACAAAGCACAT-GATAAATG
Binvadens_Kenya               AATGTATATTCCCAACAAGCCAAGTAAGTATGGAATAAAAATCCTCATGATGTGTGACAGTGGTACAAAGCACAT-GATAAATG
Binvadens_Tanzania-DES2       AATGTATATTCCCAACAAGCCAAGTAAGTATGGGATAAAAATCCTCATGATGTGTGACAGTGGTACAAAGCACAT-GATAAATG
Binvadens_Tanzania-KA         AATGTATATTCCCAACAAGCCAAGTAAGTATGGAATAAAAATCCTCATGATGTGTGACAGTGGTACAAAGCACAT-GATAAATG
Bpapayae_Australia            AATGTATATTCCCAACAAGCCAAGTAAGTATGGAATAAAAATCCTCATGATGTGTGACAGTGGTACAAAGCACAGTGAYAAATG
Bpapayae_Malaysia             AATGTATATTCCCAACAAGCCAAGTAAGTATGGAATAAAAATCCTCATGATGTGTGACAGTGGTACAAAGCACAT-GATAAATG
Bphilippinensis_Guimaras      AATGTATATTCCCAACAAGCCAAGTAAGTATGGAATAAAAATCCTCATGATGTGTGACAGTGGTACAAAGCACAT-GATAAATG
Bphilippinensis_wild1         AATGTATATTCCCAACAAGCCAAGTAAGTATGGAATAAAAATCCTCATGATGTGTGACAGTGGTACMAAGCACAT-GATAAATG
```

```
                                  1,270     1,280     1,290     1,300     1,310     1,320     1,330     1,340
Tni IFP2_piggyBac              GAATGCCTTATTTGGGAAGAGGAACACAGACCAACGGAGTACCACTCGGTGAATACTACGTGAAGGAGTTATCAAAGCCTGTGC
Bcarambolae_Bangladesh         GAATGCCTTATTTGGAAAGAGGAACTCAGACCAACGGAGTACCACTCGGTGAATACTACGTGAAGGAGTTATCAAAGCCTGTGC
Bcarambolae_French Guiana      GAATGCCTTATTTGGAAAGAGGAACTCAGACCAACGGAGTACCACTCGGTGAATACTACGTGAAGGAGTTATCAAAGCCTGTGC
Bcarambolae_Malaysia           GAATGCCTTATTTGGAAAGAGGAACTCAGACCAACGGAGTACCACTCGGTGAATACTACGTGAAGGAGTTATCAAAGCCTGTGC
Bcarambolae_Suriname           GAATGCCTTATTTGGAAAGAGGAACTCAGACCAACGGAGTACCACTCRGTGAATACTACGTGAAGGAGTTATCAAAGCCTGTGC
Bdorsalis_Kahuku               GAATGCCTTATTTGGAAAGAGGAACTCAGACCAACGGAGTACCACTCAGTGAATACTACGTGAAGGAGTTATCAAAGCCTGTGC
Bdorsalis_Hawaii-GSS           GAATGCCTTATTTGGAAAGAGGAACTCAGACCAACGGAGTACCACTCGGTGAATACTACGTGAAGGAGTTATCAAAGCCTGTGC
Binvadens_Kenya                GAATGCCTTATTTGGAAAGAGGAACTCAGACCAACGGAGTACCACTCGGTGAATACTACGTGAAGGAGTTATCAAAGCCTGTGC
Binvadens_Tanzania-DES2        GAATGCCTTATTTGGAAAGAGGAACTCAGACCAACGGAGTACCACTCGGTGAATACTACGTGAAGGAGTTATCAAAGCCTGTGC
Binvadens_Tanzania-KA          GAATGCCTTATTTGGAAAGAGGAACTCAGACCAACGGAGTACCACTCGGTGAATACTACGTGAAGGAGTTATCAAAGCCTGTGC
Bpapayae_Australia             GAATGCCTTATTTGGAAAGAGGAACTCAGACCAACGGAGTACCACTCGGTGAATACTACGTGAAGGAGTTATCAAAGCCTGTGC
Bpapayae_Malaysia              GAATGCCTTATTTGGAAAGAGGAACTCAGACCAACGGAGTACCACTCGGTGAATACTACGTGAAGGAGTTATCAAAGCCTGTGC
Bphilippinensis_Guimaras       GAATGCCTTATTTGGAAAGAGGAACTCAGACCAACGGAGTACCACTCGGTGAATACTACGTGAAGGAGTTATCAAAGCCTGTGC
Bphilippinensis_wild1          GAATGCCTTATTTGGAAAGAGGAACTCAGACCAACGGAGTACCACTCRGTGAATACTACGTGAAGGAGTTATCAAAGCCTGTGC
```

```
                            1,350     1,360     1,370     1,380     1,390     1,400     1,410     1,420
Tni IFP2_piggyBac              ACGGTAGTTGTCGTAATATTACGTGTGACCAATTGGTTCACCTCAATCCCTTTGGCAAAAAACTTACTACAAGAACCGTATAAGT
Bcarambolae_Bangladesh         ACGGTAGTTGTCGTAATATTACGTGTGACCAATTGGTTCACCTCAATCCCTTTGGCAAAAAACTTACTACAAGAACCGTACAAGT
Bcarambolae_French Guiana      ACGGTAGTTGTCGTAATATTACGTGTGACCAATTGGTTCACCTCAATCCCTTTGGCAAAAAACTTACTACAAGAACCGTACAAGT
Bcarambolae_Malaysia           ACGGTAGTTGTCGTAATATTACGTGTGACCAATTGGTTCACCTCAATCCCTTTGGCAAAAAACTTACTACAAGAACCGTACAAGT
Bcarambolae_Suriname           ACGGTAGTTGTCGTAATATTACGTGTGAAAATTGGTTCACCTCAATCCCTTTGGCAAAAAACTTACTACAAGAACCGTACAAGT
Bdorsalis_Kahuku               ACGGTAGTTGTCGTAATATTACGTGTGAAAATTGGTTCACCTCAATCCCTTTGGCAAAAAACTTACTACAAGAACCGTACAAGT
Bdorsalis_Hawaii-GSS           ACGGTAGTTGTCGTAATATTACGTGTGAAAATTGGTTCACCTCAATCCCTTTGGCAAAAAACTTACTACAAGAACCGTACAAGT
Binvadens_Kenya                ACGGTAGTTGTCGTAATATTACGTGTGAAAATTGGTTCACCTCAATCCCTTTGGCAAAAAACTTACTACAAGAACCGTACAAGT
Binvadens_Tanzania-DES2        ACGGTAGTTGTCGTAATATTACGTGTGAAAATTGGTTCACCTCAATCCCTTTGGCAAAAAACTTACTACAAGAACCGTACAAGT
Binvadens_Tanzania-KA          ACGGTAGTTGTCGTAATATTACGTGTGAAAATTGGTTCACCTCAATCCCTTTGGCAAAAAACTTACTACAAGAACCGTACAAGT
Bpapayae_Australia             ACGGTAGTTGTCGTAATATTACGTGTGAAAATTGGTTCACCTCAATCCCTTTGGCAAAAAACTTACTACAAGAACCGTACAAGT
Bpapayae_Malaysia              ACGGTAGTTGTCGTAATATTACGTGTGAAAATTGGTTCACCTCAATCCCTTTGGCAAAAAACTTACTACAAGAACCGTACAAGT
Bphilippinensis_Guimaras       ACGGTAGTTGTCGTAATATTACGTGTGAAAATTGGTTCACCTCAATCCCTTTGGCAAAAAACTTACTACAAGAACCGTACAAGT
Bphilippinensis_wild1          ACGGTAGTTGTCGTAATATTACGTGTGAAAATTGGTTCACCTCAATCCCTTTGGCAAAAAACTTACTACAAGAACCGTACAAGT
```

```
                           1,430     1,440     1,450     1,460     1,470     1,480     1,490     1,500     1,510
Tni IFP2_piggyBac              TAACCATTGTGGGAACCGTGCGATCAAACAAACGCGAGATACCGGAAGTACTGAAAAACAGTCGCTCCAGGCCAGTGGAACAT
Bcarambolae_Bangladesh         TAACCATTGTGG----------ATCAAACAAACGCGAGATACCGGAAGTACTGAAAAACAGTCGCTCCAGGCCAGTGGGACAT
Bcarambolae_French Guiana      TAACCATTGTGG----------ATCAAACAAACGCGAGATACCGGAAGTACTGAAAAACAGTCGCTCCAGGCCAGTGGGACAT
Bcarambolae_Malaysia           TAACCATTGTGG----------ATCAAACAAACGCGAGATACCGGAAGTACTGAAAAACAGTCGCTCCAGGCCAGTGGGACAT
Bcarambolae_Suriname           TAACCATTGTGG----------ATCAAACAAACGCGAGATACCGGAAGTACTGAAAAACAGTCGCTCCAGGCCAGTGGGACAT
Bdorsalis_Kahuku               TAACCATTGTGG----------ATCAAACAAACGCGAGATACCGGAAGTACTGAAAAACAGTCGCTCCAGGCCAGTGGGACAT
Bdorsalis_Hawaii-GSS           TAACCATTGTGG----------ATCAAACAAACGCGAGATACCGGAAGTACTGAAAAACAGTCGCTCCAGGCCAGTGGGACAT
Binvadens_Kenya                TAACCATTGTGG----------ATCAAACAAACGCGAGATACCGGAAGTACTGAAAAACAGTCGCTCCAGGCCAGTGGGACAT
Binvadens_Tanzania-DES2        TAACCATTGTGG----------ATCAAACAAACGCGAGATACCGGAAGTACTGAAAAACAGTCGCTCCAGGCCAGTGGGACAT
Binvadens_Tanzania-KA          TAACCATTGTGG----------ATCAAACAAACGCGAGATACCGGAAGTACTGAAAAACAGTCGCTCCAGGCCAGTGGGACAT
Bpapayae_Australia             TAACCATTGTGG----------ATCAAACAAACGCGAGATACCGGAAGTACTGAAAAACAGTCGCTCCAGGCCAGTGGGACAT
Bpapayae_Malaysia              TAACCATTGTGG----------ATCAAACAAACGCGAGATACCGGAAGTACTGAAAAACAGTCGCTCCAGGCCAGTGGGACAT
Bphilippinensis_Guimaras       TAACCATTGTGG----------ATCAAACAAACGCGAGATACCGGAAGTACTGAAAAACAGTCGCTCCAGGCCAGTGGGACAT
Bphilippinensis_wild1          TAACCATTGTGG----------ATCAAACAAACGCGAGATACCGGAAGTACTGAAAAACAGTCGCTCCAGGCCAGTGGGACAT
```

```
                                1,520     1,530     1,540     1,550     1,560     1,570     1,580     1,590
Tni IFP2_piggyBac              CGATGTTTTGTTTTGACGGACCCCTTACTCTCGTCTCATATAAACCGAAGCCAGCTAAGATGGTATACTTATTATCATCTTGTG
Bcarambolae_Bangladesh         CGATGTTTTGTTTTGACGGACCCCTTACTCTCGTCTCCTATAAACCGAAGCCAGCTAAGATGGTATACTTATTATCATCTTGTG
Bcarambolae_French Guiana      CGATGTTTTGTTTTGACGGACCCCTTACTCTCGTCTCCTATAAACCGAAGCCAGCTAAGATGGTATACTTATTATCATCTTGTG
Bcarambolae_Malaysia           CGATGTTTTGTTTTGACGGACCCCTTACTCTCGTCTCCTATAAACCGAAGCCAGCTAAGATGGTATACTTATTATCATCTTGTG
Bcarambolae_Suriname           CGATGTTTTGTTTTGACGGACCCCTTACTCTCGTCTCCAATAAACCGAAGCCAGCTAAGATGGTATACTTATTATCATCTTGTG
Bdorsalis_Kahuku               CGATGTTTTGTTTTGACGGACCCCTTACTCTCGTCTCCAATAAACCGAAGCCAGCTAAGATGGTATACTTATTATCATCTTGTG
Bdorsalis_Hawaii-GSS           CGATGTTTTGTTTTGACGGACCCCTTACTCTCGTCTCCAATAAACCGAAGCCAGCTAAGATGGTATACTTATTATCATCTTGTG
Binvadens_Kenya                CGATGTTTTGTTTTGACGGACCCCTTACTCTCGTCTCCAATAAACCGAAGCCAGCTAAGATGGTATACTTATTATCATCTTGTG
Binvadens_Tanzania-DES2        CGATGTTTTGTTTTGACGGACCCCTTACTCTCGTCTCCAATAAACCGAAGCCAGCTAAGATGGTATACTTATTATCATCTTGTG
Binvadens_Tanzania-KA          CGATGTTTTGTTTTGACGGACCCCTTACTCTCGTCTCCAATAAACCGAAGCCAGCTAAGATGGTATACTTATTATCATCTTGTG
Bpapayae_Australia             CGATGTTTTGTTTTGACGGACCCCTTACTCTCGTCTCCAATAAACCGAAGCCAGCTAAGATGGTATACTTATTATCATCTTGTG
Bpapayae_Malaysia              CGATGTTTTGTTTTGACGGACCCCTTACTCTCGTCTCCAATGAACCGAAGCCAGCTAAGATGGTATACTTATTATCATCTTGTG
Bphilippinensis_Guimaras       CGATGTTTTGTTTTGACGGACCCCTTACTCTCGTCTCCAATAAACCGAAGCCAGCTAAGATGGTATACTTATTATCATCTTGTG
Bphilippinensis_wild1          CGATGTTTTGTTTTGACGGACCCCTTACTCTCGTCTCCANTAAACCGAAGCCAGCTAAGATGGTATACTTATTATCATCTTGTG
```

```
                            1,600     1,610     1,620     1,630     1,640     1,650     1,660     1,670     1,680
Tni IFP2_piggyBac              ATGAGGATGCTTCTATCAACGAAAGTACCGGTAAACCGCAAATGGTTATGTATTATAATCAAACTAAAGGCGGAGTGGACACGC
Bcarambolae_Bangladesh         ATGAGGATGCTTCCATCAACGAAAGTACCGGTAAACCGCAAATGATTATGTATTATAACCAGACTAAAGGCGGAGTGGACACGC
Bcarambolae_French Guiana      ATGAGGATGCTTCCATCAACGAAAGTACCGGTAAACCGCAAATGATTATGTATTATAACCAGACTAAAGGCGGAGTGGACACGC
Bcarambolae_Malaysia           ATGAGGATGCTTCCATCAACGAAAGTACCGGTAAACCGCAAATGATTATGTATTATAACCAGACTAAAGGCGGAGTGGACACGC
Bcarambolae_Suriname           ATGAGGATGCTTCCATCAACGAAAGTACCGGTAAACCGCAAATGATTATGTATTATAACCAGACTAAAGGCGGAGTGGACACGC
Bdorsalis_Kahuku               ATGAGGATGCTTCCATCAACGAAAGTACCGGTAAACCGCAAATGATTATGTATTATAACCAGACTAAAGGCGGAGTGGACACGC
Bdorsalis_Hawaii-GSS           ATGAGGATGCTTCCATCAACGAAAGTACCGGTAAACCGCAAATGATTATGTATTATAACCAGACTAAAGGCGGAGTGGACACGC
Binvadens_Kenya                ATGAGGATGCTTCCATCAACGAAAGTACCGGTAAACCGCAAATGATTATGTATTATAACCAGACTAAAGGCGGAGTGGACACGC
Binvadens_Tanzania-DES2        ATGAGGATGCTTCCATCAACGAAAGTACCGGTAAACCGCAAATGATTATGTATTATAACCAGACTAAAGGCGGAGTGGACACGC
Binvadens_Tanzania-KA          ATGAGGATGCTTCCATCAACGAAAGTACCGGTAAACCGCAAATGATTATGTATTATAACCAGACTAAAGGCGGAGTGGACACGC
Bpapayae_Australia             ATGAGGATGCTTCCATCAACGAAAGTACCGGTAAACCGCAAATGATTATGTATTATAACCAGACTAAAGGCGGAGTGGACACGC
Bpapayae_Malaysia              ATGAGGATGCTTCCATCAACGAAAGTACCGGTAAACCGCAAATGATTATGTATTATAACCAGACTAAAGGCGGAGTGGACACGC
Bphilippinensis_Guimaras       ATGAGGATGCTTCCATCAACGAAAGTACCGGTAAACCGCAAATGATTATGTATTATAACCAGACTAAAGGCGGAGTGGACACGC
Bphilippinensis_wild1          ATGAGGATGCTTCCATCAACGAAAGTACCGGTAAACCGCAAATGATTATGTATTATAACCAGACTAAAGGCGGAGTGGACACGC
```

```
                                 1,690     1,700     1,710     1,720     1,730     1,740     1,750     1,760
Tni IFP2_piggyBac                TAGACCAAATGTGTTCTGTGATGACCTGCAGTAGGAAGACGAATAGGTGGCCTATGGCATTATTGTACGGAATGATAAACATTG
Bcarambolae_Bangladesh           TAGACCAAATGTGTTCTGTGATGTCCTGCAGTAGGAAGACGAATAGGTGGCCTATGGCATTATTGTACGGAATGATAAACATTG
Bcarambolae_French Guiana        TAGACCAAATGTGTTCTGTGATGTCCTGCAGTAGGAAGACGAATAGGTGGCCTATGGCATTATTGTACGGAATGATAAACATTG
Bcarambolae_Malaysia             TAGACCAAATGTGTTCTGTGATGTCCTGCAGTAGGAAGACGAATAGGTGGCCTATGGCATTATTGTACGGAATGATAAACATTG
Bcarambolae_Suriname             TAGACCAAATGTGTTCTGTGATGTCCTGCAGTAGGAAGACGAATAGGTGGCCTATGGCATTATTGTACGGAATGATAAACATTG
Bdorsalis_Kahuku                 TAGACCAAATGTGTTCTGTGATGTCCTGCAGTAGGAAGACGAATAGGTGGCCTATGGCATTATTGTACGGAATGATAAACATTG
Bdorsalis_Hawaii-GSS             TAGACCAAATGTGTTCTGTGATGTCCTGCAGTAGGAAGACGAATAGGTGGCCTATGGCATTATTGTACGGAATGATAAACATTG
Binvadens_Kenya                  TAGACCAAATGTGTTCTGTGATGTCCTGCAGTAGGAAGACGAATAGGTGGCCTATGGCATTATTGTACGGAATGATAAACATTG
Binvadens_Tanzania-DES2          TAGACCAAATGTGTTCTGTGATGTCCTGCAGTAGGAAGACGAATAGGTGGCCTATGGCATTATTGTACGGAATGATAAACATTG
Binvadens_Tanzania-KA            TAGACCAAATGTGTTCTGTGATGTCCTGCAGTAGGAAGACGAATAGGTGGCCTATGGCATTATTGTACGGAATGATAAACATTG
Bpapayae_Australia               TAGACCAAATGTGTTCTGTGATGTCCTGCAGTAGGAAGACGAATAGGTGGCCTATGGCATTATTGTACGGAATGATAAACATTG
Bpapayae_Malaysia                TAGACCAAATGTGTTCTGTGATGTCCTGCAGTAGGAAGACGAATAGGTGGCCTATGGCATTATTGTACGGAATGATAAACATTG
Bphilippinensis_Guimaras         TAGACCAAATGTGTTCTGTGATGTCCTGCAGTAGGAAGACGAATAGGTGGCCTATGGCATTATTGTACGGAATGATAAACATTG
Bphilippinensis_wild1            TAGACCAAATGTGTTCTGTGATGTCCTGCAGTAGGAAGACGAATAGGTGGCCTATGGCATTATTGTACGGAATGATAAACATTG

                                 1,770     1,780     1,790     1,800     1,810     1,820     1,830     1,840
Tni IFP2_piggyBac                CCTGCATAAATTCTTTTATTATATACAGCCATAATGTCAGTAGCAAGGGAGAAAAGGTCCAAAGTCGCAAAAAATTTATGAGAA
Bcarambolae_Bangladesh           CCTGCATAAATTCTTTTATTATATACAGCCATAATGTTAGTAGCAAGGGAGAAAAGGTTCAAAGTCGCAAAAATTTTATGAAAA
Bcarambolae_French Guiana        CCTGCATAAATTCTTTTATTATATACAGCCATAATGTTAGTAGCAAGGGAGAAAAGGTTCAAAGTCGCAAAAATTTTATGAAAA
Bcarambolae_Malaysia             CCTGCATAAATTCTTTTATTATATACAGCCATAATGTTAGTAGCAAGGGAGAAAAGGTTCAAAGTCGCAAAAATTTTATGAAAA
Bcarambolae_Suriname             CCTGCATAAATTCTTTTATTATATACAGCCATAATGTTAGTAGCAAGGGAGAAAAGGTTCAAAGTCGCAAAAATTTTATGAAAA
Bdorsalis_Kahuku                 CCTGCATAAATTCTTTTATTATATACAGCCATAATGTTAGTAGCAAGGGAGAAAAGGTTCAAAGTCGCAAAAATTTTATGAAAA
Bdorsalis_Hawaii-GSS             CCTGCATAAATTCTTTTATTATATACAGCCATAATGTTAGTAGCAAGGGAGAAAAGGTTCAAAGTCGCAAAAATTTTATGAAAA
Binvadens_Kenya                  CCTGCATAAATTCTTTTATTATATACAGCCATAATGTTAGTAGCAAGGGAGAAAAGGTTCAAAGTCGCAAAAATTTTATGAAAA
Binvadens_Tanzania-DES2          CCTGCATAAATTCTTTTATTATATACAGCCATAATGTTAGTAGCAAGGGAGAAAAGGTTCAAAGTCGCAAAAATTTTATGAAAA
Binvadens_Tanzania-KA            CCTGCATAAATTCTTTTATTATATACAGCCATAATGTTAGTAGCAAGGGAGAAAAGGTTCAAAGTCGCAAAAATTTTATGAAAA
Bpapayae_Australia               CCTGCATAAATTCTTTTATTATATACAGCCATAATGTTAGTAGCAAGGGAGAAAAGGTTCAAAGTCGCAAAAATTTTATGAAAA
Bpapayae_Malaysia                CCTGCATAAATTCTTTTATTATATACAGCCATAATGTTAGTAGCAAGGGAGAAAAGGTTCAAAGTCGCAAAAATTTTATGAAAA
Bphilippinensis_Guimaras         CCTGCATAAATTCTTTTATTATATACAGCCATAATGTTAGTAGCAAGGGAGAAAAGGTTCAAAGTCGCAAAAATTTTATGAAAA
Bphilippinensis_wild1            CCTGCATAAATTCTTTTATTATATACAGCCATAATGTTAGTAGCAAGGGAGAAAAGGTTCAAAGTCGCAAAAATTTTATGAAAA

                                 1,850     1,860     1,870     1,880     1,890     1,900     1,910     1,920     1,930
Tni IFP2_piggyBac                ACCTTTACATGAGCCTGACGTCATCGTTTATGCGTAAGCGTTTAGAAGCTCCTACTTTGAAGAGATA----TTTGCGCGATAAT
Bcarambolae_Bangladesh           ACCTTAACATGAGCCTGACGTCATCGTTTATGCGTAAGCGTTTAGAAGCTCCTACTTTGAAGAGATATGTATTTGCGCGATAAT
Bcarambolae_French Guiana        ACCTTAACATGAGCCTGACGTCATCGTTTATGCGTAAGCGTTTAGAAGCTCCTACTTTGAAGAGATATGTATTTGCGCGATAAT
Bcarambolae_Malaysia             ACCTTAACATGAGCCTGACGTCATCGTTTATGCGTAAGCGTTTAGAAGCTCCTACTTTGAAGAGATATGTATTTGCGCGATAAT
Bcarambolae_Suriname             ACCTTAACATGAGCCTGACGTCATCGTTTATGCGTAAGCGTTTAGAAGCTCCTACTTTGAAGAGATA----TTTGCGCGATAAT
Bdorsalis_Kahuku                 ACCTTAACATGAGCCTGACGTCATCGTTTATGCGTAAGCGTTTAGAAGCTCCTACTTTGAAGAGATA----TTTGCGCGATAAT
Bdorsalis_Hawaii-GSS             ACCTTAACATGAGCCTGACGTCATCGTTTATGCGTAAGCGTTTAGAAGCTCCTACTTTGAAGAGATA----TTTGCGCGATAAT
Binvadens_Kenya                  ACCTTAACATGAGCCTGACGTCATCGTTTATGCGTAAGCGTTTAGAAGCTCCTACTTTGAAGAGATA----TTTGCGCGATAAT
Binvadens_Tanzania-DES2          ACCTTAACATGAGCCTGACGTCATCGTTTATGCGTAAGCGTTTAGAAGCTCCTACTTTGAAGAGATA----TTTGCGCGATAAT
Binvadens_Tanzania-KA            ACCTTAACATGAGCCTGACGTCATCGTTTATGCGTAAGCGTTTAGAAGCTCCTACTTTGAAGAGATA----TTTGCGCGATAAT
Bpapayae_Australia               ACCTTAACATGAGCCTGACGTCATCGTTTATGCGTAAGCGTTTAGAAGCTCCTACTTTGAAGAGATA----TTTGCGCGATAAT
Bpapayae_Malaysia                ACCTTAACATGAGCCTGACGTCATCGTTTATGCGTAAGCGTTTAGAAGCTCCTACTTTGAAGAGATA----TTTGCGCGATAAT
Bphilippinensis_Guimaras         ACCTTAACATGAGCCTGACGTCATCGTTTATGCGTAAGCGTTTAGAAGCTCCTACTTTGAAGAGATA----TTTGCGCGATAAT
Bphilippinensis_wild1            ACCTTAACATGAGCCTGACGTCATCGTTTATGCGTAAGCGTTTAGAAGCTCCTACTTTGAAGAGATA----TTTGCGCGATAAT

                                 1,940     1,950     1,960     1,970     1,980     1,990     2,000     2,010
Tni IFP2_piggyBac                ATCTCTAATATTTTGCCAAATGAAGTGCCTGGTACATCAGAT-GACAGTACTGAAGAGCCAGTAATTGAAAAAACGTACTTACTG
Bcarambolae_Bangladesh           ATCTCTAATATTTTGCCAAATGAAATGCCTGGTACATCAGAAG-ACAGTACTGAAGAGCCAGTAACGAAAAAA---------CG
Bcarambolae_French Guiana        ATCTCTAATATTTTGCCAAATGAAATGCCTGGTACATCAGAAG-ACAGTACTGAAGAGCCAGTAACGAAAAAA---------CG
Bcarambolae_Malaysia             ATCTCTAATATTTTGCCAAATGAAATGCCTGGTACATCAGAAG-ACAGTACTGAAGAGCCAGTAACGAAAAAA---------CG
Bcarambolae_Suriname             ATCTCTAATATTTTGCCAAATGAAATGCCTGGTACATCAGAA-GACAGTACTGAAGAGCCAGTAACGAAAAAACGTACTTACTG
Bdorsalis_Kahuku                 ATCTCTAATATTTTGCCAAATGAAATGCCTGGTACATCAGAA-GACAGTACTGAAGAGCCAGTAACGAAAAAACGTACTTACTG
Bdorsalis_Hawaii-GSS             ATCTCTAATATTTTGCCAAATGAAATGCCTGGTACATCAGAA-GACAGTACTGAAGAGCCAGTAACGAAAAAACGTACTTACTG
Binvadens_Kenya                  ATCTCTAATATTTTGCCAAATGAAATGCCTGGTACATCAGAA-GACAGTACTGAAGAGCCAGTAACGAAAAAACGTACTTACTG
Binvadens_Tanzania-DES2          ATCTCTAATATTTTGCCAAATGAAATGCCTGGTACATCAGAA-GACAGTACTGAAGAGCCAGTAACGAAAAAACGTACTTACTG
Binvadens_Tanzania-KA            ATCTCTAATATTTTGCCAAATGAAATGCCTGGTACATCAGAAGGACAGTACTGAAGAGCCAGTAACGAAAAAACGTACTTACTG
Bpapayae_Australia               ATCTCTAATATTTTGCCAAATGAAATGCCTGGTACATCAGAA-GACAGTACTGAAGAGCCAGTAACGAAAAAACGTACTTACTG
Bpapayae_Malaysia                ATCTCTAATATTTTGCCAAATGAAATGCCTGGTACATCAGAA-GACAGTACTGAAGAGCCAGTAACGAAAAAACGTACTTACTG
Bphilippinensis_Guimaras         ATCTCTAATATTTTGCCAAATGAAATGCCTGGTACATCAGAA-GACAGTACTGAAGAGCCAGTAACGAAAAAACGTACTTACTG
Bphilippinensis_wild1            ATCTCTAATATTTTGCCAAATGAAATGCCTGGTACATCAGAA-GACAGTACTGAAGAGCCAGTAACGAAAAAACGTACTTACTG

                                 2,020     2,030     2,040     2,050     2,060     2,070     2,080     2,090     2,100
Tni IFP2_piggyBac                TACTTACTGCCCCTCTAAAATAAGGCGAAAGGCAAATGCATCGTGCAAAAAATGCAAAAAAGTTATTTGTCGAGAGCATAATAT
Bcarambolae_Bangladesh           TACTTACTGCCCCTCTAAAATAAGGCGAAAGGCAAATGCATCGTGCAAAAAATGCAAAAAAGTTATTTGTCGAGAGCATAATAT
Bcarambolae_French Guiana        TACTTACTGCCCCTCTAAAATAAGGCGAAAGGCAAATGCATCGTGCAAAAAATGCAAAAAAGTTATTTGTCGAGAGCATAATAT
Bcarambolae_Malaysia             TACTTACTGCCCCTCTAAAATAAGGCGAAAGGCAAATGCATCGTGCAAAAAATGCAAAAAAGTTATTTGTCGAGAGCATAATAT
Bcarambolae_Suriname             TACTTACTGCCCCTCTAAAATAAGGCGAAAGGCAAATGCATCGTGCAAAAAATGCAAAAAAGTTATTTGTCGAGAGCATAATAT
Bdorsalis_Kahuku                 TACTTACTGCCCCTCTAAAATAAGGCGAAAGGCAAATGCATCGTGCAAAAAATGCAAAAAAGTTATTTGTCGAGAGCATAATAT
Bdorsalis_Hawaii-GSS             TACTTACTGCCCCTCTAAAATAAGGCGAAAGGCAAATGCATCGTGCAAAAAATGCAAAAAAGTTATTTGTCGAGAGCATAATAT
Binvadens_Kenya                  TACTTACTGCCCCTCTAAAATAAGGCGAAAGGCAAATGCATCGTGCAAAAAATGCAAAAAAGTTATTTGTCGAGAGCATAATAT
Binvadens_Tanzania-DES2          TACTTACTGCCCCTCTAAAATAAGGCGAAAGGCAAATGCATCGTGCAAAAAATGCAAAAAAGTTATTTGTCGAGAGCATAATAT
Binvadens_Tanzania-KA            TACTTACTGCCCCTCTAAAATAAGGCGAAAGGCAAATGCATCGTGCAAAAAATGCAAAAAAGTTATTTGTCGAGAGCATAATAT
Bpapayae_Australia               TACTTACTGCCCCTCTAAAATAAGGCGAAAGGCAAATGCATCGTGCAAAAAATGCAAAAAAGTTATTTGTCGAGAGCATAATAT
Bpapayae_Malaysia                TACTTACTGCCCCTCTAAAATAAGGCGAAAGGCAAATGCATCGTGCAAAAAATGCAAAAAAGTTATTTGTCGAGAGCATAATAT
Bphilippinensis_Guimaras         TACTTACTGCCCCTCTAAAATAAGGCGAAAGGCAAATGCATCGTGCAAAAAATGCAAAAAAGTTATTTGTCGAGAGCATAATAT
Bphilippinensis_wild1            TACTTACTGCCCCTCTAAAATAAGGCGAAAGGCAAATGCATCGTGCAAAAAATGCAAAAAAGTTATTTGTCGAGAGCATAATAT
```

```
                                2,110     2,120     2,130     2,140     2,150     2,160     2,170     2,180
Tni IFP2_piggyBac               TGATATGTGCCAAAGTTGTTTCTGACTGACTAATAAGTATAATTTGTTTCTATTATGTATAAGTTAAGCTAATTACTTATTTTA
Bcarambolae_Bangladesh          TGATATTTGCCAAAGTTGTTTCTGACTGACTAATAAGTAAAATTTGTTTCTAATATGTATAAGATATGCTAATTATTTATTTTA
Bcarambolae_French Guiana       TGATATTTGCCAAAGTTGTTTCTGACTGACTAATAAGTAAAATTTGTTTCTAATATGTATAAGATATGCTAATTATTTATTTTA
Bcarambolae_Malaysia            TGATATTTGCCAAAGTTGTTTCTGACTGACTAATAAGTAAAATTTGTTTCTAATATGTATAAGATATGCTAATTATTTATTTTA
Bcarambolae_Suriname            TGATATTTGCCAAAGTTGTTTCTGACTGACTAATAAGTAAAATTTGTTTCTAATATGTATAAGATATGCTAATTATTTATTTTA
Bdorsalis_Kahuku                TGATATTTGCCAAAGTTGTTTCTGACTGACTAATAAGTAAAATTTGTTTCTAATATGTATAAGATATGCTAATTATTTATTTTA
Bdorsalis_Hawaii-GSS            TGATATTTGCCAAAGTTGTTTCTGACTGACTAATAAGTAAAATTTGTTTCTAATATGTATAAGATATGCTAATTATTTATTTTA
Binvadens_Kenya                 TGATATTTGCCAAAGTTGTTTCTGACTGACTAATAAGTAAAATTTGTTTCTAATATGTATAAGATATGCTAATTATTTATTTTA
Binvadens_Tanzania-DES2         TGATATTTGCCAAAGTTGTTTCTGACTGACTAATAAGTAAAATTTGTTTCTAATATGTATAAGATATGCTAATTATTTATTTTA
Binvadens_Tanzania-KA           TGATATTTGCCAAAGTTATTTCTGACTGACTAATAAGTAAAATTTGTTTCTAATATGTATAAGATATGCTAATTATTTATTTTA
Bpapayae_Australia              TGATATTTGCCAAAGTTGTTTCTGACTGACTAATAAGTAAAATTTGTTTCTAATATGTATAAGATATGCTAATTATTTATTTTA
Bpapayae_Malaysia               TGATATTTGCCAAAGTTGTTTCTGACTGACTAATAAGTAAAATTTGTTTCTAATATGTATAAGATATGCTAATTATTTATTTTA
Bphilippinensis_Guimaras        TGATATTTGCCAAAGTTGTTTCTGACTGACTAATAAGTAAAATTTGTTTCTAATATGTATAAGATATGCTAATTATTTATTTTA
Bphilippinensis_wild1           TGATATTTGCCAAAGTTGTTTCTGACTGACTAATAAGTAAAATTTGTTTCTAATATGTATAAGATATGCTAATTATTTATTTTA

                                2,190     2,200     2,210     2,220     2,230     2,240     2,250     2,260
Tni IFP2_piggyBac               TAATACAACATGACTGTTTTTAAAGTACAAAATAAGTTTATTTTTGTAAAAGAGAGAATGTTTAAAAGTTTTGTTACTTTATAG
Bcarambolae_Bangladesh          TAATACAACATGACTGATTTCATAGTACAAAATAAGTTTATT-TTGTAAAAGAGAGAATGTTTAAAAGTTTTGTTACTTTATAG
Bcarambolae_French Guiana       TAATACAACATGACTGATTTCATAGTACAAAATAAGTTTATT-TTGTAAAAGAGAGAATGTTTAAAAGTTTTGTTACTTTATAG
Bcarambolae_Malaysia            TAATACAACATGACTGATTTCATAGTACAAAATAAGTTTATT-TTGTAAAAGAGAGAATGTTTAAAAGTTTTGTTACTTTATAG
Bcarambolae_Suriname            TAATACAACACGACTGATTTCATAGTACAAAATAAGTTTATT-TTGTAAAAGAGAGAATGTTTAAAAGTTTTGTTACTTTATAG
Bdorsalis_Kahuku                TAATACAACACGACTGATTTCATAGTACAAAATAAGTTTATT-TTGTAAAAGAGAGAATGTTTAAAAGTTTTGTTACTTTATAG
Bdorsalis_Hawaii-GSS            TAATACAACACGACTGATTTCATAGTACAAAATAAGTTTATT-TTGTAAAAGAGAGAATGTTTAAAAGTTTTGTTACTTTATAG
Binvadens_Kenya                 TAATACAACACGACTGATTTCATAGTACAAAATAAGTTTATT-TTGTAAAAGAGAGAATGTTTAAAAGTTTTGTTACTTTATAG
Binvadens_Tanzania-DES2         TAATACAACACGACTGATTTCATAGTACAAAATAAGTTTATT-TTGTAAAAGAGAGAATGTTTAAAAGTTTTGTTACTTTATAG
Binvadens_Tanzania-KA           TAATACAACACGACTGATTTCATAGTACAAAATAAGTTTATT-TTGTAAAAGAGAGAATGTTTAAAAGTTTTGTTACTTTATAG
Bpapayae_Australia              TAATACAACACGACTGATTTCATAGTACAAAATAAGTTTATT-TTGTAAAAGAGAGAATGTTTAAAAGTTTTGTTACTTTATAG
Bpapayae_Malaysia               TAATACAACACGACTGATTTCATAGTACAAAATAAGTTTATT-TTGTAAAAGAGAGAATGTTTAAAAGTTTTGTTACTTTATAG
Bphilippinensis_Guimaras        TAATACAACACGACTGATTTCATAGTACAAAATAAGTTTATT-TTGTAAAAGAGAGAATGTTTAAAAGTTTTGTTACTTTATAG
Bphilippinensis_wild1           TAATACAACACGACTGATTTCATAGTACAAAATAAGTTTATT-TTGTAAAAGAGAGAATGTTTAAAAGTTTTGTTACTTTATAG

                                2,270     2,280     2,290     2,300     2,310     2,320     2,330     2,340     2,350
Tni IFP2_piggyBac               AAGAAATTTTGAGTTTTTGTTT---TTTTTTAATAAATAAATAAACATAAATAAATTGTTTGTTGAATTTATTATTAGTATGTA
Bcarambolae_Bangladesh          AAGAAGTTTTAAGTTTTTGTTTT--TTTTTTAATGAATAAATAAACATAAATAAATTATTTGTTGAATTTATTATTAGTATGTA
Bcarambolae_French Guiana       AAGAAGTTTTAAGTTTTTGTTTT--TTTTTTAATGAATAAATAAACATAAATAAATTATTTGTTGAATTTATTATTAGTATGTA
Bcarambolae_Malaysia            AAGAAGTTTTAAGTTTTTGTTTT--TTTTTTAATGAATAAATAAACATAAATAAATTATTTGTTGAATTTATTATTAGTATGTA
Bcarambolae_Suriname            AAGAAGTTTTAAGTTTTTGTTTTT-TTTTTTAATGAATAAATAAACATAAATAAATTATTTGTTGAATTTATTATTAGTATGTA
Bdorsalis_Kahuku                AAGAAGTTTTAAGTTTTTGTTTTT-TTTTTTAATGAATAAATAAACATAAATAAATTATTTGTTGAATTTATTATTAGTATGTA
Bdorsalis_Hawaii-GSS            AAGAAGTTTTAAGTTTTTGTTTTTTTTTTTNAATGAATAMATAAACATAAATAAATTATTTGTTGAATTTATTATTAGTATGTA
Binvadens_Kenya                 AAGAAGTTTTAAGTTTTTGTTTTTTTTTTTTAATGAATAAATAAACATAAATAAATTATTTGTTGAATTTATTATTAGTATGTA
Binvadens_Tanzania-DES2         AAGAAGTTTTAAGTTTTTGTTTTTTTTTTTTAATGAATAAATAAACATAAATAAATTATTTGTTGAATTTATTATTAGTATGTA
Binvadens_Tanzania-KA           AAGAAGTTTTAAGTTTTTGTTTT--TTTTTTAATGAATAAATAAACATAAATAAATTATTTGTTGAATTTATTATTAGTATGTA
Bpapayae_Australia              AAGAAGTTTTAAGTTTTTGTTTTT-TTTTTTAATGAATAAATAAACATAAATAAATTATTTGTTGAATTTATTATTAGTATGTA
Bpapayae_Malaysia               AAGAAGTTTTAAGTTTTTGTTTTT-TTTTTTAATGAATAAATAAACATAAATAAATTATTTGTTGAATTTATTATTAGTATGTA
Bphilippinensis_Guimaras        AAGAAGTTTTAAGTTTTTGTTTTTTTTTTNNAATGAATAAATAAACATAAATAAATTATTTGTTGAATTTATTATTAGTATGTA
Bphilippinensis_wild1           AAGAAGTTTTAAGTTTTTGTTTTT-TTTTTNAATGAATAAATAAACATAAATAAATTATTTGTTGAATTTATTATTAGTATGTA

                                2,360     2,370     2,380     2,390     2,400     2,410     2,420     2,430
Tni IFP2_piggyBac               AGTGTAAATATAATAAAACTTAATATCTATTCAAATTAATAAATAAACCTCGATATACAGACCGATAAAACACATGCGTCAATT
Bcarambolae_Bangladesh          AGTGTAAATATAATAAAACGTAATATCTATTCAAATTAATAAATAAACCTCGATATACAGACCGATAAAACACATGCGTCAATT
Bcarambolae_French Guiana       AGTGTAAATATAATAAAACGTAATATCTATTCAAATTAATAAATAAACCTCGATATACAGACCGATAAAACACATGCGTCAATT
Bcarambolae_Malaysia            AGTGTAAATATAATAAAACGTAATATCTATTCAAATTAATAAATAAACCTCGATATACAGACCGATAAAACACATGCGTCAATT
Bcarambolae_Suriname            AGTGTAAATATAATAAAACGTAATATCTATTCAAATTAATAAATAAACCTCGATATACAGACCGATAAAACACATGCGTCAATT
Bdorsalis_Kahuku                AGTGTAAATATAATAAAACGTAATATCTATTCAAATTAATAAATAAACCTCGATATACAGACCGATAAAACACATGCGTCAATT
Bdorsalis_Hawaii-GSS            AGTGTAAATATAATAAAACGTAATATCTATTCAAATTAATAAATAAACCTCGATATACAGACCGATAAAACACATGCGTCAATT
Binvadens_Kenya                 AGTGTAAATATAATAAAACGTAATATCTATTCAAATTAATAAATAAACCTCGATATACAGACCGATAAAACACATGCGTCAATT
Binvadens_Tanzania-DES2         AGTGTAAATATAATAAAACGTAATATCTATTCAAATTAATAAATAAACCTCGATATACAGACCGATAAAACACATGCGTCAATT
Binvadens_Tanzania-KA           AGTGTAAATATAATAAAACGTAATATCTATTCAAATTAATAAATAAACCTCGATATACAGACCGATAAAACACATGCGTCAATT
Bpapayae_Australia              AGTGTAAATATAATAAAACGTAATATCTATTCAAATTAATAAATAAACCTCGATATACAGACCGATAAAACACATGCGTCAATT
Bpapayae_Malaysia               AGTGTAAATATAATAAAACGTAATATCTATTCAAATTAATAAATAAACCTCGATATACAGACCGATAAAACACATGCGTCAATT
Bphilippinensis_Guimaras        AGTGTAAATATAATAAAACGTAATATCTATTCAAATTAATAAATAAACCTCGATATACAGACCGATAAAACACATGCGTCAATT
Bphilippinensis_wild1           AGTGTAAATATAATAAAACGTAATATCTATTCAAATTAATAAATAAACCTCGATATACAGACCGATAAAACACATGCGTCAATT

                                2,440     2,450     2,460     2,470     2,480  2,487
Tni IFP2_piggyBac               TTACGCATGATTATCTTTAACGTACGTCACAATATGATTATCTTTCTAGGG
Bcarambolae_Bangladesh          TTACGCATGATTATCTTTAACGTACGTCACAATATGATTATCTTTCTAGGG
Bcarambolae_French Guiana       TTACGCATGATTATCTTTAACGTACGTCACAATATGATTATCTTTCTAGGG
Bcarambolae_Malaysia            TTACGCATGATTATCTTTAACGTACGTCACAATATGATTATCTTTCTAGGG
Bcarambolae_Suriname            TTACGCATGATTATCTTTAACGTACGTCACAATATGATTATCTTTCTAGGG
Bdorsalis_Kahuku                TTACGCATGATTATCTTTAACGTACGTCACAATATGATTATCTTTCTAGGG
Bdorsalis_Hawaii-GSS            TTACGCATGATTATCTTTAACGTACGTCACAATATGATTATCTTTCTAGGG
Binvadens_Kenya                 TTACGCATGATTATCTTTAACGTACGTCACAATATGATTATCTTTCTAGGG
Binvadens_Tanzania-DES2         TTACGCATGATTATCTTTAACGTACGTCACAATATGATTATCTTTCTAGGG
Binvadens_Tanzania-KA           TTACGCATGATTATCTTTAACGTACGTCACAATATGATTATCTTTCTAGGG
Bpapayae_Australia              TTACGCATGATTATCTTTAACGGACGTCACAATATGATTATCTTTCTAGGG
Bpapayae_Malaysia               TTACGCATGATTATCTTTAACGTACGTCACAATATGATTATCTTTCTAGGG
Bphilippinensis_Guimaras        TTACGCATGATTATCTTTAACGTACGTCACAATATGATTATCTTTCTAGGG
Bphilippinensis_wild1           TTACGCATGATTATCTTTAACGTACGTCACAATATGATTATCTTTCTAGGG
```

Figure S1. Clustal Omega multiple nucleotide sequence alignment of the full-length *T. ni* IFP2 *piggyBac* transposon sequence (2,472 bp) and *pBac*$^{Bd\text{-}Kah}$ homologs (2,419-2,459 bp) isolated from 13 strains from five *Bactrocera dorsalis* complex species. The IFP2 *piggyBac* open reading frame sequence extends from nt 335 to nt 2122 relative to the consensus sequence (numbered above). Note colored indels and nucleotide substitutions in *pBac*$^{Bd\text{-}Kah}$ homologs compared to IFP2 *piggyBac*, and common and variant mutations in the taxa synonymized with *B. dorsalis* (*B. invadens*, *B. papayae*, and *B. phillipinensis* strains) compared to the *B. carambolae* strains. The sequence alignment was subjected to a Maximum-Likelihood phylogenetic analysis (Fig. 4) and GenBank accession identifiers for sequences are presented in Table 1.

A

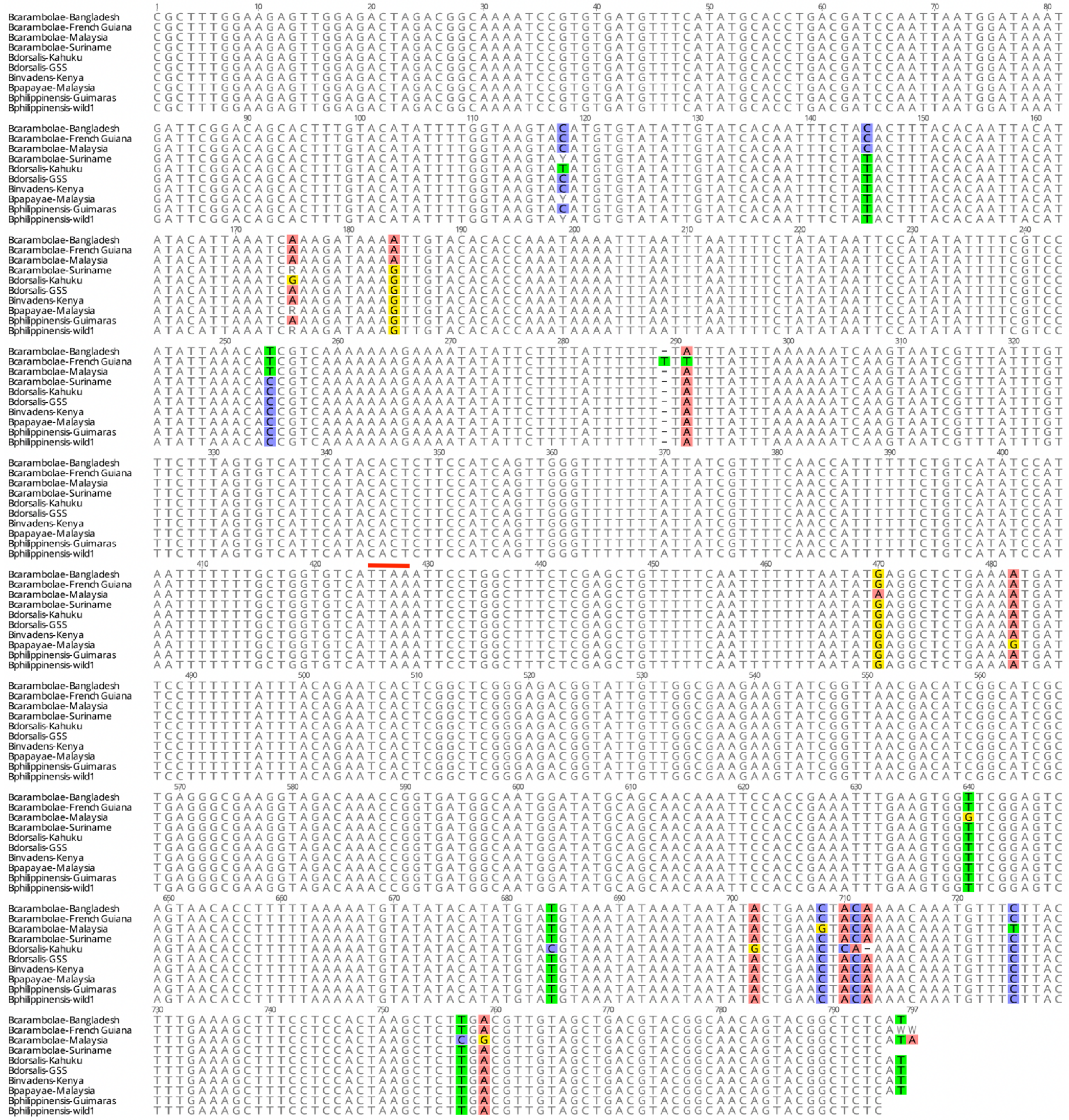

B

	Bcar-Bangladesh	Bcar-Fr Guiana	Bcar-Malaysia	Bcar-Suriname	Bdor-Kahuku	Bdor-GSS	Binv-Kenya	Bpap-Malaysia	Bphil-Guimaras
Bcarambolae-Bangladesh									
Bcarambolae-French Guiana	99.7								
Bcarambolae-Malaysia	99.2	98.9							
Bcarambolae-Suriname	99.5	99.2	98.7						
Bdorsalis-Kahuku	98.7	98.4	98.1	99.2					
Bdorsalis-GSS	99.6	99.3	98.9	99.9	99.1				
Binvadens-Kenya	99.6	99.3	98.9	99.9	99.1	100			
Bpapayae-Malaysia	99.4	99.1	98.6	99.7	99.1	99.7	99.7		
Bphilippinensis-Guimaras	99.6	99.4	98.9	99.9	99.1	100	100	99.7	
Bphilippinensis-wild1	99.5	99.2	98.7	99.9	99.2	99.9	99.9	99.7	99.9

Figure S2. A) Clustal Omega multiple sequence alignment of the insertion site nucleotide sequences adjacent to the *pBac*$^{Bd\text{-}Kah}$ homologs from indicated species and strains (single sequence presented for species having identical sequence in all strains), and B) percent identity matrix between the sequences. Genomic sequences 5' and 3' to the transposon insertion were isolated by PCR using the AH513 and AH514 genomic primers, respectively, with internal *piggyBac* primers. The genomic sequences were linked after deleting the *piggyBac* sequence and a single duplicated TTAA tetranucleotide insertion site (red bar), which yielded 793 bp to 797 bp genomic sequences.
